# A New Diterpenoid of Indonesian *Scoparia dulcis* Linn: Isolation and Cytotoxic Activity against MCF-7 and T47D Cell Lines

**DOI:** 10.3390/molecules28165960

**Published:** 2023-08-09

**Authors:** Hasnawati Hasnawati, Subagus Wahyuono, Ratna Asmah Susidarti, Djoko Santosa, Arfan Arfan

**Affiliations:** 1 Faculty of Pharmacy, Universitas Halu Oleo, Kendari 93232, Indonesia; arfan09@uho.ac.id; 2 Doctoral Program in Pharmaceutical Science, Faculty of Pharmacy, Universitas Gadjah Mada, Yogyakarta 55281, Indonesia; 3 Department of Pharmaceutical Biology, Faculty of Pharmacy, Universitas Gadjah Mada, Yogyakarta 55281, Indonesia; djoko5346@ugm.ac.id; 4 Department of Chemistry Pharmaceutical, Faculty of Pharmacy, Universitas Gadjah Mada, Yogyakarta 55281, Indonesia; ratna_asmah@ugm.ac.id

**Keywords:** *Scoparia dulcis*, 2α-hydroxyscopadiol, cytotoxic, MCF-7, T47D, molecular docking

## Abstract

*Scoparia dulcis* Linn plays an important role in treatment because it contains active compounds that are proven to have a variety of activities, including cytotoxicity on various cancer cells. The objective of this study is to isolate and identify the cytotoxic compounds in the ethyl acetate fraction of *Scoparia dulcis*, observe cell cycle inhibition and induction of apoptosis in vitro, and carry out molecular studies using in silico studies. A new diterpene compound was isolated from the ethyl acetate fraction of *Scoparia dulcis* L. of Indonesian origin. Chromatographic methods were used to isolate the compound, spectroscopic methods were used to elucidate its structure, and these data were compared with those reported in the literature. The compound was tested for its cytotoxic activity against two breast cancer cells (MCF-7 and T47D). The results of the isolated compound showed a cytotoxic effect on MCF-7 and T47D breast cancer cells at IC_50_ 70.56 ± 1.54 and <3.125 ± 0.43 µg/mL, respectively. The compound inhibited the growth of MCF-7 and T47D breast cancer cells and the accumulation of cells in the G1 phases, and it induced apoptosis. Based on a spectroscopic analysis, the isolated compound was identified as 2α-hydroxyscopadiol, which is a new diterpenoid. A docking study revealed that the isolate’s hydroxyl groups are essential for interacting with crucial residues on the active sites of the ER and PR and caspase-9. The isolate inhibits ER and PR activity with binding energies of −8.2 kcal/mol and −7.3 kcal/mol, respectively. In addition, the isolate was also able to induce apoptosis through the activation of the caspase-9 pathway with an affinity of −9.0 kcal/mol. In conclusion, the isolated compound from *S. dulcis* demonstrated anticancer activity based on in vitro and in silico studies.

## 1. Introduction

Breast cancer is one of the main causes of death. In 2018, this disease showed the highest percentage of new cases, namely, 42.1%, and the percentage of deaths was 17.0% (World Health Organization, 2019 [1]). Breast cancer deaths generally occur due to delays in handling and treating sufferers, so the cancer is already in an advanced stage or is difficult to cure. Given these facts, the development of a comprehensive therapy to treat breast cancer is needed to reduce the number of deaths. The development of new cancer therapies from medicinal plants like chemotherapy is ongoing, and many efforts have been made to find new active compounds from medicinal plants for chemotherapy to overcome resistance, increase efficacy, and reduce the toxic effects of chemotherapy [2]. The advantage of using natural compounds produced by medicinal plants is that they have relatively few side effects when used correctly and appropriately [3]. The discovery of compounds from medicinal plants and natural producers is generally carried out by screening natural materials, extracting and isolating secondary metabolites, purifying the compounds contained, finding their chemical structures, and then testing them with an appropriate biological assay system to obtain lead compounds [4].

One of the medicines that can be used for cancer therapies is *Scoparia dulcis* Linn. This plant is a member of Scrophulariaceae. *Scoparia dulcis* Linn (*S. dulcis*) is wide spread in America, Africa, Europe, and Asia. The plant has been broadly used for eras in southern China, India, Brazil, Paraguay, and Nigeria. Traditional Chinese medicine believes that *S. dulcis* has stomachic, diuretic, antitussive, warmth-clearing, and toxin-soaking effects. In ethnomedicine, *S. dulcis* is used to treat gastric problems, edema, liver sicknesses, and breathing sicknesses [5]. According to the current literature, many terpenoid compounds are found in *S. dulcis*. The tetracyclic diterpenoids include scopadulcic acid A and B, dulcinol/scopadulciol are the labdane-derived diterpenoids (scopadulane) [6], and 4-*epi*-scopadulcic acid B, dulcidiol, iso-dulcinol [7], and scopadulcic acid C [8] are also found. Some of these chemical constituents have antioxidant and cytotoxic [9,10,11,12], anticancer [6,7,13,14,15,16,17,18,19], and antiproliferative [14] activities. The metabolites of *S. dulcis* contain groups of phenolic compounds; diterpenoids, triterpenoids, alkaloids, flavonoids, steroids, and other aliphatics.

This study is a continuation of previous research [20], in which our previous results revealed that ethyl acetate fractions in the ethanolic extract of *S. dulcis* exhibited cytotoxic activity against MCF-7 and T47D breast cancer cell lines. So, in this study, the active compound was isolated from the ethyl acetate fraction of *S. dulcis*, and its cytotoxic activity was determined against MCF-7 and T47D breast cancer cells. Several previous studies stated that various compounds from *S. dulcis* Linn have cytotoxic activity in many cells such as human stomach cancer cells [7], prostate cancer cells [16], and HeLa cancer cells [14]. Ref. [21] reported three new diterpene isolated from this plant: scopadulcic acid A (**1**), scopadulcic acid B (**2**), dulcinol/scopadulciol (**3**). Compound (**3**) was reported to show cytotoxic, antiproliferative, and apoptotic antitumor and anticancer activity against AGS human gastric adenocarcinoma cells [15]. 

In this study, a new diterpene compound (compound **1**) was discovered that had never been reported before in this plant. An evaluation was conducted utilizing an in silico approach with molecular docking to understand the molecular interactions of this compound with estrogen receptor (ER), progesterone receptor (PR), and caspase-9 targets. These targets are crucial, especially in MCF-7 and T47D breast cancer cells. Therefore, this approach gives a deeper insight into the molecular mechanism of this new diterpene compound as an anticancer agent.

## 2. Results

**Figure 1:**
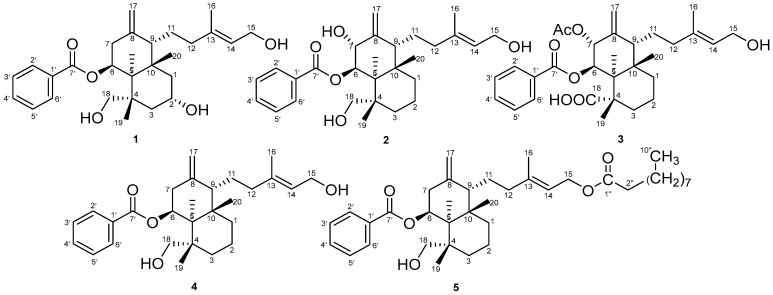
Chemical structures of 2α-hydroxyscopadiol (**1**), 7 α-hydroxyscopadiol (**2**) [22], 4-*epi*-7α-*O*-acetylscoparic acid A (**3**) [22], scopadiol (**4**) [23], and scopadiol decanoate (**5**) [21].

Continuing our previous work, we report the isolation of a new diterpene from the plant *Scoparia dulcis*. The isolation and identification of the ethyl acetate fraction from aerial parts of *S. dulcis* yielded a new compound that had not been reported before. This compound was identified as 2-hydroxyscopadiol (compound (**1**)), which was identified using UV-Vis, IR, LC-MS/MS, and 1D and 2D NMR. We then compared these data with data from a study that we collected from the literature, in which we found four structures similar to compound (**1**) (Figure 1). The chemical structure of the compound obtained from that study was (**2**) 7α-hydroxyscopadiol [22], (**3**) 4-*epi*-7α-*O*-acetylscoparic acid A [22], (**4**) scopadiol [23], and (**5**) scopadiol decanoate [21].

### 2.1. Properties of 2α-Hydroxyscopadiol (Compound ***1***)

**Figure 2:**
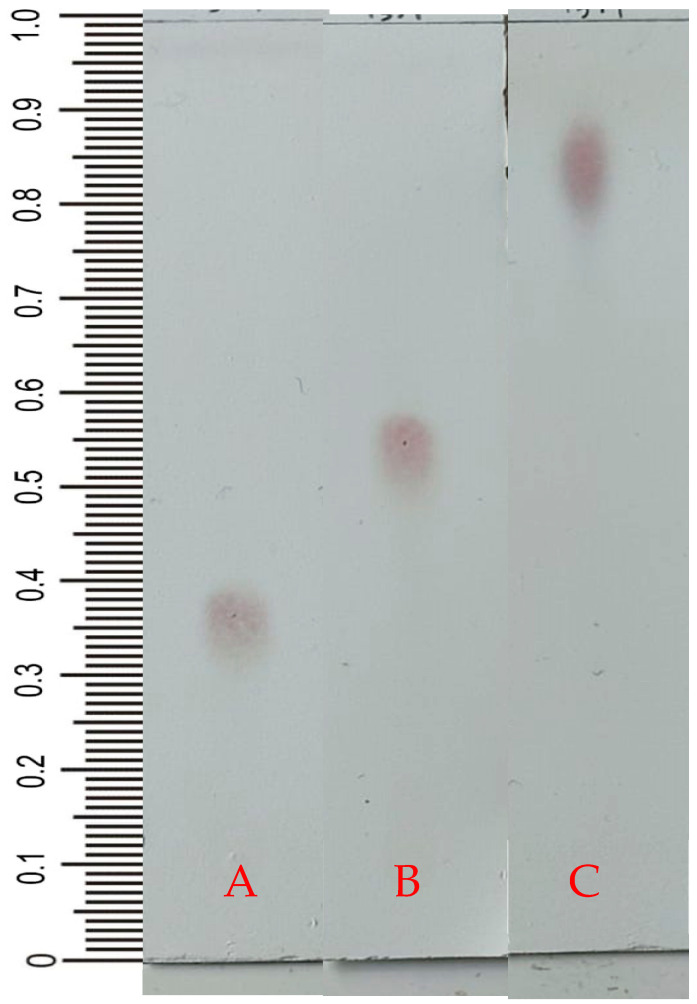
TLC profile with vanillin sulfate spray from the isolated 13.1 compound. Mobile phase: (**A**) n-hexane:ethyl acetate (2:3); (**B**) Chloroform:ethylacetate (1:3); and (**C**) n-hexane:ethanol (1:2).

White powder compound; 25 mg; mp 176–178 °C, Rf = 0.29 (n-hexane:ethyl acetate, 2:3); Rf = 0.56 (chloroform:ethyl acetate, 1:3); Rf = 0.81 (n-hexane:etanol) (Figure 2), muffle under UV lamp (254 nm; UV-visible λ_max_: 248 nm (log ε) 3.54; IR (KBr) ν_max_ cm^−1^: 3429.97; 2925.98; 2854.01; 1602.95; 1384.23; 1356.65; 1111.79; 712.93. Table 1 and Table 2 contain the ^1^H-NMR (500 MHz, CDCl_3_) and ^13^C-NMR (125 MHz, CDCl_3_) spectra data, respectively. The LC-MS/MS TOF MS^E^ (100–1200) 6 eV ESI^+^-Low CE (BPI) showed *m*/*z* 465.2644 [M + Na]^+^ with neutral mass (Da) 442.2719. *m*/*z* (% intensity) of molecular ion: 465 (51) ([M + Na]^+^) and *m*/*z* of possible fragments: 321 (100) ([M − C_7_H_5_O_2_]^+^); 303 (83) ([M − C_7_H_5_O_2_ − H_2_O]^+^); 285 (54) ([M − C_7_H_5_O_2_ − 2H_2_O]^+^). The molecular formula was C_27_H_38_O_5_. Compound (**1**) was identified as 2α-hydroxyscopadiol.

### 2.2. Thin-Layer Chromatography

The isolation and identification of *S. dulcis* compounds in the ethyl acetate fraction (20 g) were carried out using a vacuum liquid chromatography (VLC) column, thin layer chromatography (TLC), and purification as well as preparative thin layer chromatography (PTLC). The isolates were then examined for chromatogram profiles using TLC under UV light 254, 366 nm, and a spray reaction with vanillin sulfate.

### 2.3. Spectroscopic Analysis

The ^1^H and ^13^C NMR (Table 1 and Table 2, respectively) signals of compound (**1**) were found to be similar to those of scopadiol (**4**) [23], scopadiol decanoate (**5**) [21], and 7α-hydroxyscopadiol (**2**) [22]. The data for compounds (**2**), (**3**), (**4**), and (**5**) were obtained from the study that we collected from the literature.

#### COSY and HMBC Correlations of Compound (**1**)

**Figure 3:**
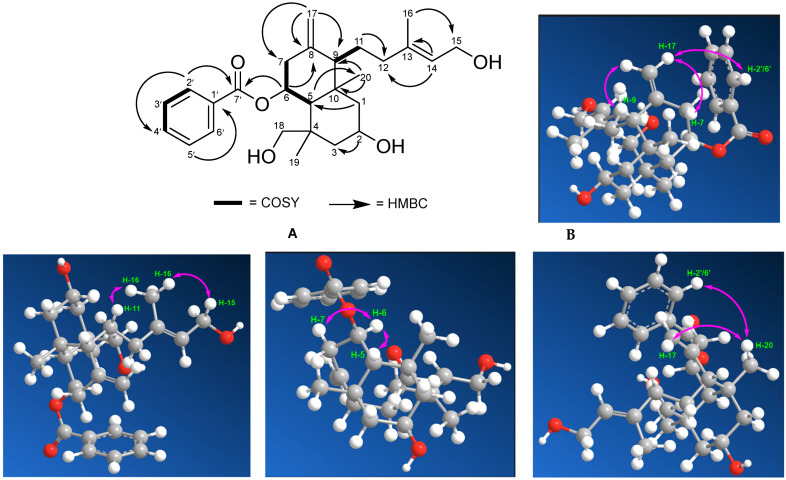
The COSY and HMBC (**A**) and (**B**) correlations showing the NOESY interactions in compound (**1**).

The COSY and HMBC correlations (A) and (B) showing NOESY interactions in compound (**1**) are displayed in Figure 3.

### 2.4. Cytotoxic Activity

**Figure 4:**
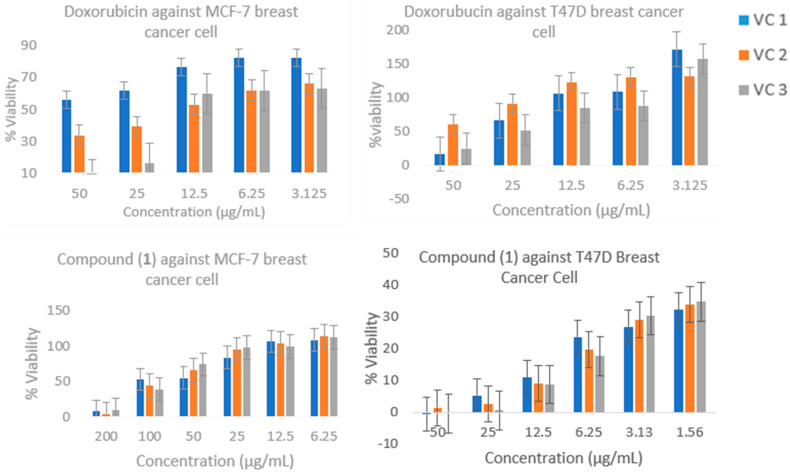
The % viability value for compound (**1**) and doxorubicin. All data are presented as mean ± SD, *n* = 3 and VC 1: variation of concentration 1; VC 2: variation of concentration 2; and VC 3: variation of concentration 3.

A cytotoxic test was performed to obtain an overview of the activity of the sample on the inhibition of cancer cells. The parameter used was the inhibition concentration of 50% (IC_50_), a concentration that inhibits cells by 50% of the cell population. The IC_50_ value was calculated from the results of a linear regression between log concentration and the percentage of cell viability. The calculated IC_50_ values are presented in Table 3 and Figure 4.

### 2.5. Cell Cycle and Apoptosis

**Figure 5:**
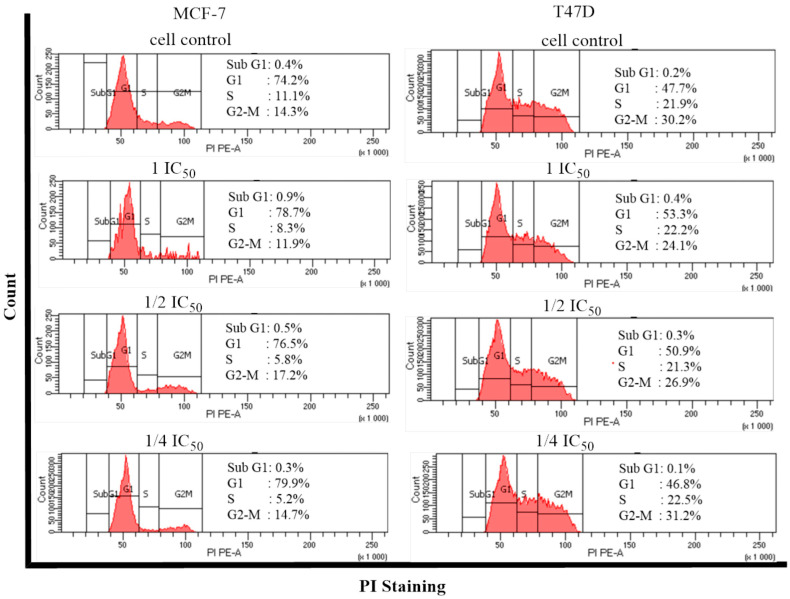
Histogram profile of the cell cycle for compound (**1**) against MCF-7 and T47D breast cancer cells and the control.

**Figure 6:**
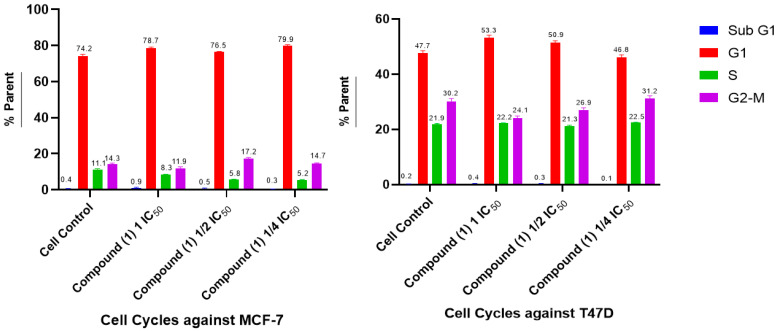
Cell cycle distribution after compound (**1**) treatment using flow cytometry against MCF-7 and T47D breast cancer cells.

Cell cycle inhibition and apoptosis induction tests were carried out on compound (**1**). The analysis was performed using the flow cytometry method to determine the percentage of living cells in each phase of the cell cycle. The results for the cell cycle are shown in Figure 5 and Figure 6.

**Figure 7:**
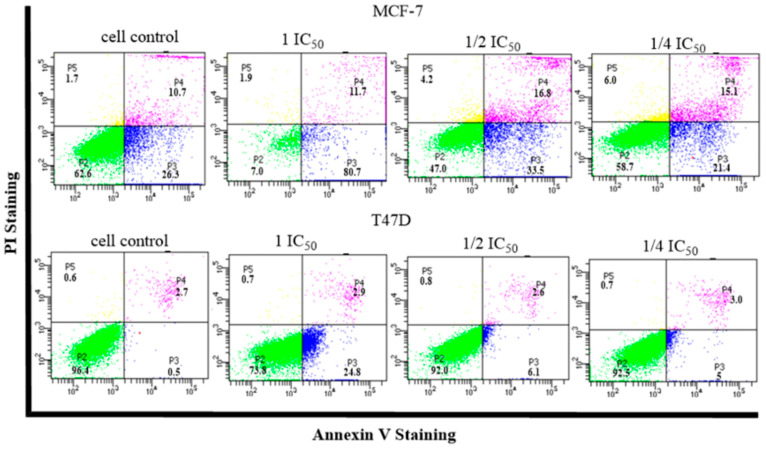
Histogram profile for apoptosis induction following treatment using compound (**1**) against MCF-7 and T47D breast cancer cell and cell control.

**Figure 8:**
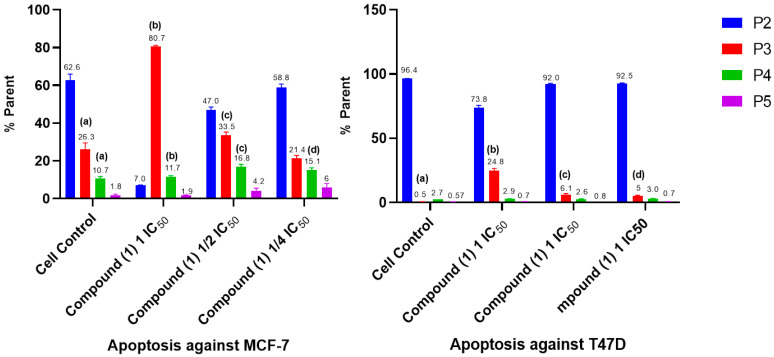
Effect of apoptosis induction after compound 1 treatment. Error bars represent the standard deviation (*n* = 3) and quadrant P2 indicates living cells. P3: early apoptosis, P4: late apoptosis, P5: necrosis. Different letters (a–d) indicate significant differences based on an ANOVA followed by a Tukey post hoc test (*p* < 0.05).

Induction of apoptosis was observed to determine the cell mechanism underlying the treatment of MCF-7 and T47D breast cancer cells with compound (**1**), which were incubated for 24 h. The method used in this study was the detected Annexin V method using flow cytometry, which allowed for observing the induction of apoptosis that occurs in cells following treatment. Annexin V is a member of a family of phospholipid-binding proteins that are strongly negatively charged on the cell membrane. Cell death resulting from apoptosis or necrosis can be differentiated using propidium iodide (PI) staining intercalation with DNA [24]. The results for the induction of apoptosis obtained using flow cytometry are shown in Figure 7 and Figure 8.

### 2.6. Molecular Docking Study

**Figure 9:**
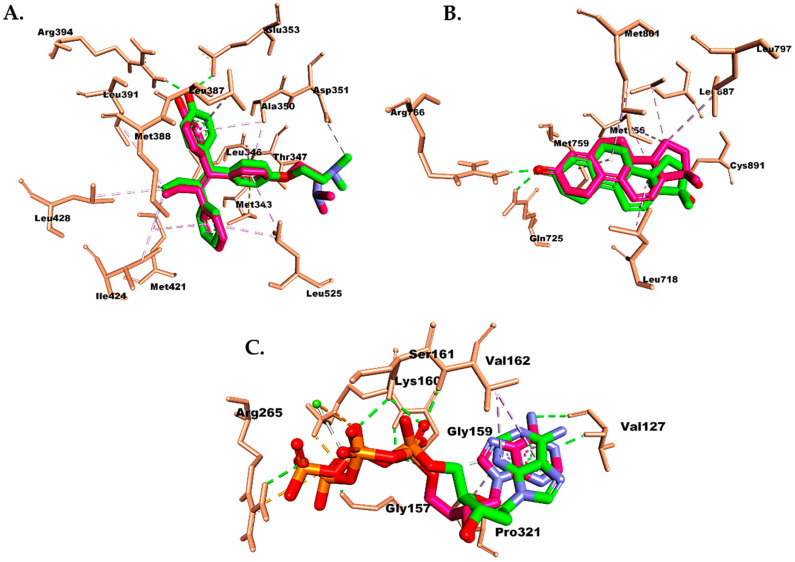
Superimpose and the interaction between the co-crystal ligand (green) and the docked pose (pink) of (**A**) TMX in the ER, (**B**) R18 in the PR, and (**C**) DTP in caspase-9.

The preferred binding mechanisms of the compounds to the receptors were predicted using molecular docking analyses, which revealed binding affinity and residual amino acid interactions. Specifically, our attention was directed toward characterizing the engagement between important amino acid residues within the estrogen receptor (ER) and progesterone receptor (PR) and caspase-9. These specific residues play a pivotal role in enhancing the stability of the interaction between the compounds and the receptors. To evaluate the simulation, we performed a redocking of the co-crystal ligand onto its receptor and measured the RMSD. A lower RMSD value signifies a higher level of accuracy in the docking parameters’ ability to replicate the conformation of the co-crystal ligand, as observed using X-ray crystallography. The best conformation of the co-crystal ligands (TMX, R18, and DTP) to their receptors are shown in Figure 9.

The redocking analysis of the co-crystal ligand to the binding site of the receptors revealed a pose similar to the reported X-ray crystallography, with an RMSD of 1.057 Å for TMX in ER (Figure 9A), 0.927 Å for R18 in PR (Figure 9B), and 1.887 Å for DTP in caspase-9 (Figure 9C). In this study, the co-crystal ligand exhibited stronger affinities toward the receptors compared with doxorubicin and compound (**1**), with energies of −9.8 kcal/mol for TMX, −11.5 kcal/mol for R18, and −9.9 kcal/mol for DTP (Table 4).

Meanwhile, the isolate showed a stronger affinity than doxorubicin, with an energy of −8.2 kcal/mol for the ER and −7.3 kcal/mol for the PR. However, compound (**1**) exhibited a weaker affinity toward caspase-9, with an energy of −9.0 kcal/mol (Table 4).

**Figure 10:**
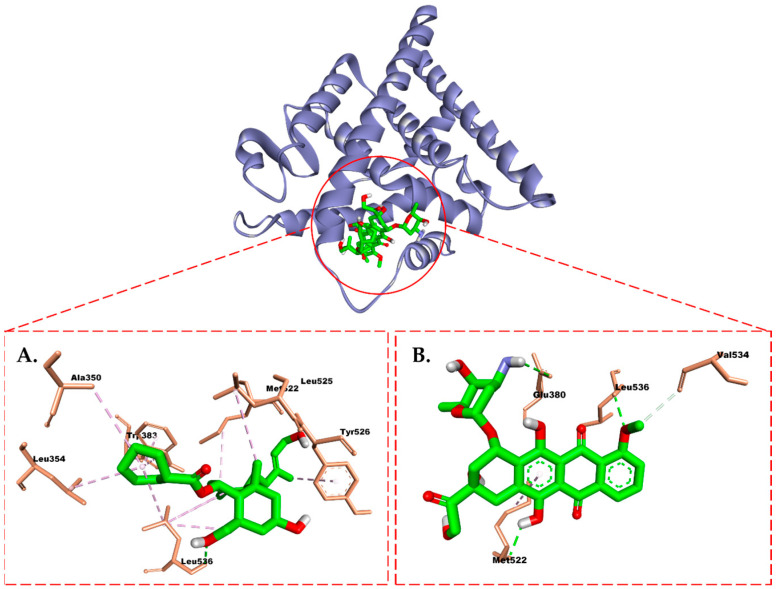
Molecular interaction between (**A**) compound (**1**) and (**B**) doxorubicin and the estrogen receptor.

Compound (**1**) exhibited a hydrogen bond (H-bond) in the ER between its hydroxyl group and Leu536. This H-bond was also observed in doxorubicin but with the additional involvement of Glu830 and Met522 residues (Figure 10). The TMX co-crystal (tamoxifen) formed two distinct H-bond (Glu353 and Arg394) with the isolate compounds. In addition, the resulting hydrophobic interactions were similar, involving Leu387, Leu391, Leu346, Ala350, Leu428, Met388, Leu525, Met421, and Ile424 (Figure 9A). Interestingly, the methyl and cyclohexane groups in compound (**1**) contribute to a non-polar region, leading to hydrophobic interactions at the active site of the ER that differ from those of tamoxifen. Specifically, these interactions involve Trp383, Tyr526, and Met522 residues.

**Figure 11:**
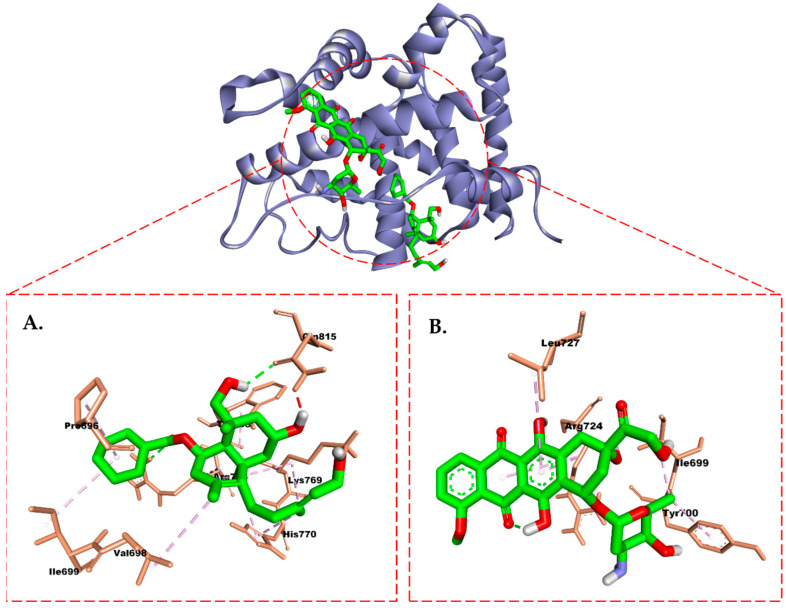
Molecular interaction between (**A**) compound (**1**) and (**B**) doxorubicin and the progesterone receptor.

In PR, the isolate exhibited the presence of two H-bonds with residues Arg766 and Gln815, formed by the hydroxyl group of the compound (**1**), as well as Arg766 and Gln725 from R18 (Figure 11). Binding to Arg766 is considered important for inhibiting the activity of PR. In contrast, doxorubicin did not form any H-bond with the PR. In addition, the hydrophobic interaction demonstrated by doxorubicin was weaker than the isolate, involving residues Leu727, Tyr700, Ile699, and Arg724. This interaction explains the displacement of doxorubicin from the active site of PR, as it differs significantly from the hydrophobic interaction shown by R18 toward residues Leu718, Met756, Cys891, Leu797, Leu887, Met759, and Met801.

**Figure 12:**
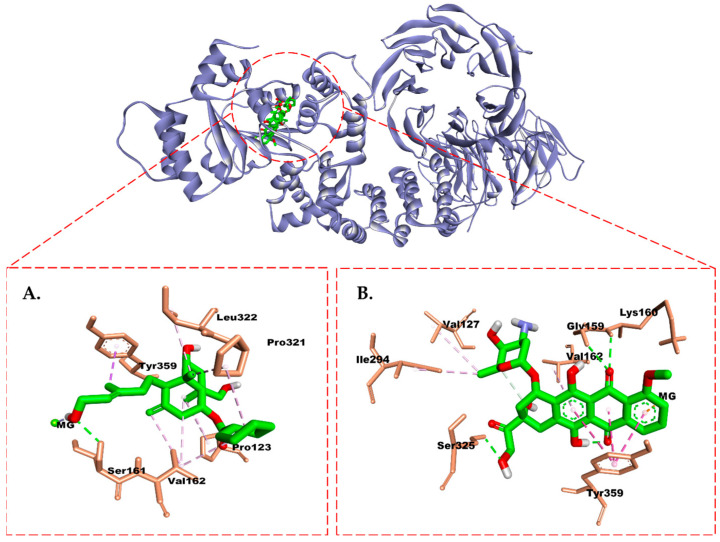
Molecular interaction between (**A**) compound (**1**) and (**B**) doxorubicin and caspase-9.

Finally, in the case of caspase-9, DTP and doxorubicin exhibited nearly equal affinity. In DTP, H-bonds were observed with Gly157, Ser161, Gly159, Val162, Val127, and Arg265. Additionally, hydrophobic interactions (Val162 and Pro321) and interactions with Mg^2+^ ions at the active site of caspase-9 were also observed (Figure 12). The H-bond interaction shown by doxorubicin was similar to that of DTP but differed in residues Ser325 and Lys160. Interestingly, although the isolated compound had a weaker affinity than doxorubicin, it still fell within a range of energy that is strong enough to bind to caspase-9. The isolate only formed one H-bond with a similar Ser161 residue as in DTP. Furthermore, the isolate was capable of forming hydrophobic interactions with residues Tyr359, Leu322, Val162, Pro321, and Pro123, as well as interactions with Mg^2+^ ions at the active site of caspase-9, which is similar to DTP and doxorubicin.

## 3. Discussion

This study aims to isolate cytotoxic compounds from the ethyl acetate fraction of *Scoparia dulcis* Linn against two breast cancer cell lines: MCF-7 and T47D. In this study, a new compound was isolated that had never been reported in this plant.

a. Isolation and Identification of Compound (**1**)

The choice of a system for compound isolation is largely determined by the character of a compound that can be predicted from the best chromatogram data with the stationary phase-mobile phase-specific spotting of the fraction obtained. The bioassay process begins with testing an extract to confirm the presence or absence of cytotoxic activity. The extract that has the strongest activity proceeds to the fractionation stage. The separation process is continued on the most active fraction while the less active fraction is set aside. The separation process and cytotoxic test are repeated until a pure compound is obtained.

The isolation of compound 1 was carried out using a combination of bioassay methods and a phytochemical approach. This was performed so that the compound isolation could be directed according to the specified activity, namely, as a cytotoxic agent. In this study, the bioassay was carried out in vitro by conducting a cytotoxicity test on MCF-7 and T47D breast cancer cells using a 3-(4,5-dimethylthiazol-2-yl)-2,5-diphenyltetrazolium bromide (MTT) assay. Using this method, the chemical structures of organic compounds are determined spectroscopically, which can be performed if the organic compound is obtained in a pure state.

The structure of organic compounds can be determined using the spectroscopic method. The spectroscopic method is currently the best method because it can be performed in a short time using a small number of samples. Mass spectra and NMR data provide molecular formula information (C_x_H_y_O_z_) and the degree of unsaturation, also known as double bond equivalents. While the NMR spectra, infrared, and UV/Vis will provide information on the presence of functional groups, NMR spectra will show the parts of the structure forming these compounds. One-dimensional NMR spectroscopy data, infrared, mass, and UV/Vis spectra are used to identify a target compound using compounds that have been previously discovered (known compounds). But for organic compounds without a previously known chemical structure (new compounds), these data are not enough to explain the chemical structure, so spectral data are needed such as 2D NMR spectra, namely, HMQC, HMBC, and COSY.

Compound **1** was obtained as a white, amorphous powder, and the results of TLC showed that the isolated compound (compound (**1**)) has a purity profile with three different mobile phases. The TLC plate sprayed with vanillin sulfate showed one spot at Rf = 0.29 (n-hexane:ethyl acetate, 2:3); at Rf = 0.56 (chloroform:ethyl acetate, 1:3); and at Rf = 0.81 (n-hexane:etanol) (Figure 2), and the melting point test obtained a temperature of 176–178 °C. These results indicate that the isolated compound is pure. The TLC chromatogram indicates the presence of terpenoid compounds in pure compounds, which are shown with a color change to a red-purplish color after heating.

The ultraviolet (UV) spectrum exhibited a λmax of 248 nm and a log ε of 3.54, which predicted that the molecule structure consisted of two to three possible conjugated double bonds. The infrared (IR) spectrum showed a peak at 3429.97 cm^−1^ that indicated a hydroxyl group (OH). In addition, the absorbances at 2925.98 cm^−1^ and 2854.01 cm^−1^ characterized the aliphatic C-H stretch. At the same time, the aromatic carbon double bond (C=C) group exhibited a strong peak at 1602.95 cm^−1^. The absorbances at 1384.23 cm^−1^ and 1356.65 cm^−1^ showed C-H bending, and the absorbance at 1111.79 cm^−1^ indicated the presence of a C-O stretch. The LC-MS/MS (ESI^+^) spectrum showed that the molecular formula of the compound (**1**) was C_27_H_38_O_5_, and it exhibited a molecular ion peak at *m*/*z* 465 (M + Na)^+^. The ion peak at *m*/*z* 321 indicated the release of benzoate (-C_7_H_5_O_2_) groups. Meanwhile, the ion peak at *m*/*z* 303 characterized the release of the dihydrogen oxide group, and the ion peak at *m*/*z* 285 lost two times dihydrogen oxide.

Based on the known number of C and H atoms from the NMR data, the data obtained indicate that there are five O atoms in this molecule, including three hydroxyl groups (OH); therefore, the chemical formula of this compound is C_27_H_38_O_5_. The index of hydrogen deficiency (IHD) of this compound is nine. From the IHD data, it can be estimated that this compound has one aromatic ring, one carbonyl group, two aliphatic rings, and two aliphatic double bonds. This compound consists of two components, namely, diterpene (aliphatic) and benzoate (aromatic) components. This is based on the range of chemical shifts in the carbon–carbon compounds in this compound, which are either in the aromatic area (>6 ppm) or the aliphatic area (<6 ppm).

The presence of an aromatic ring in this compound is indicated by C-1′ (δc = 130.7) to C-6′ (δc = 129.9), while the carbonyl carbon is at a chemical shift of 166.4 ppm (C-7′), which is characteristic of carbonyl esters. The aromatic part of this compound is a mono-substituted aromatic ring characterized by the presence of three aromatic proton signals, two of which represent two protons each (H-2′6′ and H-3′5′). The three aromatic proton signals have a coupling constant (J) between 7 and 8 Hz, which indicates ortho coupling with each other. The aliphatic part of this compound is a diterpene group with a total of 20 carbons. The NMR spectrum indicates that there are three methyl groups (C-16, C-19, and C-20), which bind three singlet protons each. The presence of two double bonds is indicated by two pairs of carbon with a chemical shift above 100 ppm. One of the two pairs of double-bond carbons consists of a quaternary C (C-8) and CH_2_ (C-17), where C-17 binds a proton integrating two protons (4.71 ppm), while the other double-bond pair consists of a quaternary C (C-13) and CH (C-14). Furthermore, the diterpene moiety of this compound contains six CH_2_ groups (in addition to C-17), of which two (C-15 and C-18) are methylene groups attached to a hydroxyl group (OH), characterized by proton-downfield protons (>3 ppm). In addition, there are two more methine carbons (CH) that bind O, namely, C-2 (δc = 75.2), which also binds the OH group, and C-6 (δc = 71.6), which binds to the O ester in the benzoate group.

Based on studies from the literature, we found that the ^1^H and ^13^C NMR (Table 1 and Table 2, respectively) signals of compound (**1**) were similar to those of Scopadiol (**4**) [23], scopadiol decanoate (**5**) [21], and 7α-hydroxyscopadiol (**2**) [22]. Significant differences were detected in C-2 between compounds (**1**) and (**4**). The ^13^C NMR spectrum showed a signal of 27 carbons (Table 2). There were downfield signals for H-2 (δ_H_ = 4.20, m) and C-2 (δ_C_ = 75.2) of compound **1** when compared with the H-2 (-) and C-2 (-) signals of scopadiol (**4**), scopadiol decanoate (**5**), and 7α-hydroxyscopadiol (**2**). H-7 (δ_H_ = 4.16, d, J = 2.4) and C-7 (δc = 75.7) of compound **2** had a downfield signal when compared with H-7 (δ_H_ = 2.32 (2H, dd, J = 5, J = 15) and C-7 (δ_C_ = 36.9) of compound (**1**), (**4**), and (**5**). There were downfield signals for H-15 (δ_H_ = 3.66 m; 3.50 dt (5;10)) and C-15 (δc = 65.7) of compound (**1**) when compared with the H-15 and C-15 signals of scopadiol decanoate (**5**). Compound (**5**) had a fatty acid chain substituent at C-15 instead of C-18, with the presence of a carboxymethylene and a methyl group at C-2″ and C-10″ (δ_H_ = 2.31, t, J = 7.2; δc = 38.4) and (δ_H_ = 0.90, t, J = 7.2 ; δc = 15.0), respectively.

From this data comparison, compound (**1**) lost a methylene signal and showed a methine signal carrying additional oxygen. The COSY and HMBC correlations (Figure 3A) between H-2 (δ_H_ = 4.20, m) and C-1 and C-3 (δc = 38.1) indicated that the hydroxy group is attached to the C-2. H-1 (δ_H_ = 1.13 m; 1.58 m) was corelated with H-5 (δ_H_ = 2.06, d, J = 2.5). The COSY H-5 (δ_H_ = 2.06, d, J = 2.5) was corelated with H-6 (δ_H_ = 5.60 s); H-6 corelated with H-7 (δ_H_ = 2.32 m); H-9 (δ_H_ = 1.80 m) was correlated with H-11 (δ_H_ = 1.74, m); and H-3′ (δ_H_ = 7.43, t, d = 7.5) was corelated with H-2′ (δ_H_ = 8.01, t, d = 7.5) and H-4′ (δ_H_ = 7.54, t, d = 7.5). The HMBC (Figure 2A) H-1 (δ_H_ = 1.13 m; 1.58 m) were correlated with C-5 (δc = 41.1); H-5 (δ_H_ = 2.06, d, J = 2.5) were corelated with C-20 (δc = 26.0); and H-20 (δ_H_ = 1.44, s) were corelated with C-9 and C-10. The correlations between H-17 (δ_H_ = 4.71, s) and C-7 (δc = 36.9), C-8 (δc = 144.4), and C-9 (δc = 57.5) and between H-14 (δ_H_ = 5.12, d, J = 10)) and C-12 (δc = 31.5) and C-13 (Δ^13^) (δc = 148.9) provide information about the position of the aliphatic double bond in the diterpenes. H-6 (δ_H_ = 5.60 s) and H-2′ (δ_H_ = 8.01, t, d = 7.5) were corelated with C-7′, the carbonyl group from ester. In addition, the position of the methyl group was also indicated by the correlation between H-16 (δ_H_ = 1.24, s) and C-15 (δc = 65.8).

Thus, the planar structure of compound (**1**), the configurations of the hydroxymethyl group at the C-4 position, and the hydroxy group at the C-2 position were determined to be α-oriented, respectively, using the NOESY spectrum (Figure 3B). There were correlations between H-20 (δ_H_ = 1.44, s) and H-2′6′ (δ_H_ = 8.01) and between H-17 (δ_H_ = 4.71, s) and H-17 (δ_H_ = 4.71, s) and H-7 (δ_H_ = 2.33, m) and H-9 (δ_H_ = 1.80,m). H-7 ((δ_H_ = 2.33, m) was corelated with H-6 ((δ_H_ = 5.60, s) and H-6 ((δ_H_ = 5.60, s) was correlated with H-5 (δ_H_ = 2.06, d, J = 2.5), suggesting that H-6 and H-5 are α-oriented and H-9 is β-oriented. This suggests that the hydroxy group at the C-2 position and the hydroxymethyl group at the C-4 position are both α-oriented. This is because the hydroxy group is in an equatorial and more stable position compared with when it is in an axial position. In addition, it is bulky and has lower energy. Therefore, compound (**1**) is referred to as 2α-hydroxyscopadiol.

b. Cytotoxic Test

A material or compound that can be said to have anticancer activity must be proven using various tests, one of which is a cytotoxic test. A material or compound that has good cytotoxic activity during in vitro or in vivo tests can be said to have potential as a material or compound anticancer. The cytotoxic test examines a substance or chemical compound using a cell culture system to determine the effect of materials or compounds on the viability of the cells used. The search for anticancer compounds can be carried out using various methods at the cellular and molecular levels with in vitro studies. Testing cell viability is one of the cytotoxicity test methods used to measure the proportion of cell life after a traumatic event.

The parameter used for the cytotoxic test was the IC_50_ value. IC_50_ values indicate the concentration value that results in the inhibition of cell proliferation by 50% and the potential toxicity of a compound to cells. This value is a benchmark for conducting kinetic tests. The IC_50_ value can show the potential of a compound as being cytotoxic. As the IC_50_ value increases, the compound toxicity decreases. The results of a cytotoxicity test on the target organ provide direct information about changes that occur specifically in cell functions.

Studies on the proliferation, viability, and mortality of deep cell research on anticancer compounds require accurate quantification of the cell numbers in any culture. One method that is widely used is the 3-([4,4-dimethylthiazol-2-yl]-2,5-diphenyl-tetrazolium bromide) (MTT) reaction. The MTT reaction is a cellular reduction reaction based on the breakdown of salts. The yellow MTT tetrazolium turns into colored formazan crystals with a purplish-blue color. Mitochondrial reductase enzymes in living cells are able to break down MTT to become formazan crystals. The formazan crystals formed can be dissolved with a certain solvent, and then the absorbance can be read using a microplate reader at a wavelength of 560–570 nm [25]. Greater absorbance values indicate that the tested material or compound is non-toxic.

A cytotoxic test was performed to obtain an overview of the activity of the sample on the inhibition of cancer cells. The IC_50_ value was calculated from the results of a linear regression between log concentration and the percentage of cell viability. A cytotoxicity test was performed on compound (**1**) to determine its effect on the viability of MCF-7 and T47D cells. The calculated IC_50_ values are presented in Table 3. In vitro anticancer cytotoxic activity was grouped according to the IC_50_ values: “very strong cytotoxic”: IC_50_ < 10 µg/mL; “strong cytotoxic” IC_50_ is 10–100 µg/mL; and “moderately cytotoxic”: IC_50_ is 100–500 µg/mL [26]. Based on cell viability, compound (**1**) treatment showed a linear relationship between concentration and cell inhibitory activity. Compound (**1**) (Table 3) showed cytotoxic activity values against MCF-7 and T47D cancer cells with IC_50_ values of 70.56 ± 1.54 and <3.125 ± 0.43 µg/mL, respectively. The ANOVA results showed that there was a significant difference when compared with the IC_50_ value of the control doxorubicin, with IC_50_ values of 16.62 ± 5.85 and 35.08 ± 3.14 µg/mL, respectively, and a significance of *p* < 0.05 (Table 3, Figure 5). Therefore, this compound showed cytotoxic properties against MCF-7 and T47D breast cancer cells. Compound (**1**) had high selectivity for T47D cells but was not selective for MCF-7 cells when compared with doxorubicin, which had high selectivity for MCF-7 cells but was not selective for T47D. The SI value was determined by comparing the IC_50_ values for the Vero cell line with IC_50_ values for MCF-7 and T47D cancer cells. A compound is said to have high selectivity for cancer cells if the SI value ≥ 3 [27,28]. Table 3 shows that compound 1 had an SI value > 3 for T47D cells when compared with doxorubicin (SI < 3), which suggests that compound **1** has high selectivity for T47D cancer cells but not for doxorubicin. Therefore, in the future, it is very possible that this compound can be developed as an anticancer agent for breast cancer. Several studies in the literature reported that scopadiol (compound (**4**)) shows cytotoxic activity against SCL, SCL-6, SCL-3 7′ 6, SCL-9, KATO-3, NUG-4, stomach cancer cells with ED_50_ = 8.9 to 46.0 µM [7], and AGS gastric adenocarcinoma cells [15] and the DU-145 prostate cancer cell line [16]. Furthermore, 4-*epi*-7α-*O*-acetylscoparic acid A (compound (**3**)) showed potent agonistic activities against PPAR-γ, with EC_50_ 0.9 ± 0.3 µM.

c. Cell Cycle and Apoptosis

The cytotoxicity test with the MTT assay was only able to explain the activity of compounds in inhibiting the growth of cancer cells using the IC_50_ parameter. IC_50_ values and morphological changes cannot identify the cause of cell death. Therefore, a cell cycle inhibition test and induction of apoptosis were carried out using the flow cytometry method. The cell cycle is a regulator of cell proliferation and growth, including cell division after DNA damage. Cycle cells regulate the transition from the quiescence phase (G_0_) to cell proliferation, and continuity in genetic transcription is ensured by various checkpoints. Cell cycle inhibition is a cell survival mechanism that works by repairing DNA damage. Activation of apoptotic signals will occur if the checkpoint is not functioning and if DNA repair has not been completed [29].

Observing of the cell cycle is one method to determine the distribution of cell populations at each stage of cell development. A cell cycle analysis can be carried out to determine the effect of a material or compound on inhibiting cell growth at certain stages of the cell cycle [30]. The distribution of cell populations at each stage of the cell cycle can be determined using flow cytometry. The cells to be tested are stained with a fluorescent dye, then the cells are passed at a capillary rate of thousands of cells per second and directed to a laser beam or equivalent monochromatic light source. The fluorescence characteristics of each cell are then collected and, with the help of software, the distributed fluorescence of each cell is displayed in the form of a histogram. This tool can also be used to calculate the DNA content using cell staining. The most widely used cell dye in cell cycle analysis is propidium iodide (PI) [31]. Propidium iodide is used to color each phase because it is capable of interacting with DNA [32]. Flow cytometry can detect each phase of the cell cycle based on the number of chromosomes in each phase (G1, S, and G2/M). The G1 phase has 2*n* (diploid) chromosomes, the S phase undergoes replication in preparation for entering the G2 phase so that the number of chromosome sets is between 2*n* and 4*n*, and the G2 phase has 4*n* (2 diploid cells) chromosomes.

The results of this study (Figure 5 and Figure 6) showed that control cells undergo a distribution of cells in the sub-G1, G1, S, and G2-M phases. Compound (**1**) in MCF-7 cells causes accumulation in the G1 phase at a concentration of 1 IC_50_, ½ IC_50,_ and ¼ IC_50_ and the G2-M phase at a concentration of ½ IC_50_, with an average percentage of each concentration in the G1 phase of 78.4, 76.5, and 79.8%, respectively, compared with the cell control of 74.2%, and in phase G2-M: 17.2% compared with the control of 14.3%. Whereas in T47D cells (Figure 6), compound (**1**) accumulated in the G1 phase at a concentration of 1 IC_50_ and ½ IC_50_, with a percentage of 53.3 and 50.9% compared with the control of 47.7%. From these data, it can be seen that compound (**1**) is capable of increasing the percentage of cell cycles in the G1 phases in both MCF-7 and T47D cells. Thus, this compound is capable of inducing G1 arrest in both MCF-7 and T47D cells. G1 phase inhibition (G1 arrest) occurs via the checkpoint 2 (Chk2) activation pathway and the p53 activation pathway. Phosphorylation of mouse double minute 2 (MDM2) and MDM4 will activate p53, which will then initiate p21 expression. Increased p21 expression can inhibit Cdk-activating kinase (CAK), resulting in Cdk/cyclin E and Cdk complexes with other cyclins becoming inactive in the G1 phase. Thus, there is a buildup of the cell population in G1, which then undergo apoptosis [33].

Cell accumulation occurs in the sub-G1 phase. Cells in this phase are hypodiploid with low DNA content. This is a possibility due to DNA fragmentation, which is a sign of cell death. Accumulation in this phase indicates the induction of cell death. The percentage of sub-G1 phase cells indicates the population of apoptotic cells. From the G0 phase (resting diploid state) to the G1 phase, the number of sets of chromosomes is 2*n*. During the DNA synthesis (S) phase, the number of sets is 2*n* to 4*n*. In the G2 phase, the cell has 4 sets of chromosomes. At the end of mitosis, two cells will be produced. Furthermore, the cell enters the diploid G0 phase, which is ready to carry out the cell cycle or remain in the G0 phase [32].

When DNA damage is detected, the G2/M checkpoint works to stop the cell process before entering the mitotic phase [34] so that during this condition, the cell will carry out the DNA repair process. If the DNA repair process is successful, the cell will enter the cell cycle stage, whereas failure to repair will lead the cell to apoptosis [35]. We know that most cell cycles are in the G1/S or G2/M stages [30], and p53 plays a role in regulating the G2/M phase transition to the process of apoptosis, and DNA damage will trigger cell cycle arrest or apoptosis depending on the degree of damage.

Apoptosis is programmed cell death. Under normal circumstances, if there is damage at the cellular level that cannot be repaired, then the cell will undergo apoptosis, but this does not occur in cancer cells. Therefore, apoptosis is one of the targets of efforts to control cancer cells. By restoring gene function and/or inducing the genes involved in the process, apoptosis is expected to restore normal cell function [36]. While the cell undergoes apoptosis, changes occur on the surface plasma membrane. One of the changes that occur in the plasma membrane is the translocation of phosphatidylserine (PS) from the inner to the outer layers of the membrane plasma so that PS is exposed to the outer surface of the cell. PS exposure on the outside of the plasma membrane can be detected using Annexin V [37,38]. Annexin V is a binding protein of phospholipids whose work depends on Ca^2+^ ions. Annexin V has a high affinity with PS, and thus, it can be used as a probe that is sensitive to PS exposure on the outer layer of the plasma membrane. Therefore, Annexin V has become an excellent probe for detecting apoptotic cells. PS exposure also occurs in necrotic cells. To differentiate apoptotic and necrotic cells in general, Annexin V is used together with PI. In this test, Annexin V will bind to PS from apoptotic and necrotic cells, whereas PI will only bind to DNA derived from necrosis cells [31].

The induction of apoptosis was observed to determine the cell mechanism underlying treatment with compound (**1**) against MCF-7 and T47D breast cancer cells, which were incubated for 24 h. The method used in this study was the detected Annexin V method using flow cytometry, which identified the induction of apoptosis that occurred in cells given the treatment. Annexin V is a member of a family of phospholipid-binding proteins that are strongly negatively charged on the cell membrane. Cell death resulting from apoptosis or necrosis can be differentiated using propidium iodide (PI) staining intercalation with DNA [39]. The results for the induction of apoptosis using flow cytometry and the percentage of cell death after undergoing treatment caused by apoptosis or necrosis are shown in Figure 7.

Most of the MCF-7 and T47D cells that received compound (**1**) treatment were able to increase the number of cells that underwent early and late apoptosis: early apoptosis at concentrations 1 IC_50_ and 1/2 I IC_50_ of 80.7 and 33.5%, respectively, against 26.5% of the cell control (MCF-7); and (T47D) at concentrations of 1 IC_50_, 1/2 IC_50_, and 1/4 IC_50_ of 24.8, 6.1, and 5%, respectively, against 0.5% of the cell control. Late apoptosis showed an increase of 11.7, 16.8, and 15.1% against the cell control from 10.7% (MCF-7) and T47D from 2.9 (1 IC_50_) and 3.0% (1/2 IC_50_) against 2.7% of the cell control. Based on the ANOVA analysis (Figure 8), there was a significant difference between each concentration in compound (**1**) and the cell control with a significant value of *p* < 0.05. This value indicates that compound (**1**) has the ability to induce apoptosis of MCF-7 and T47D cells in early and late apoptosis against MCF-7 and T47D.

The mechanism underlying cell apoptosis is very complex. Basically, apoptosis is divided into 4 stages, namely, the existence of a death signal (from inside or outside the cell), stage regulation of transduction pathways (signal transduction and induction of associated apoptotic genes), the implementation stages of apoptosis (DNA degradation and cell disassembly), and phagocytosis [31]. T47D cells are breast cancer cells of characteristic caspase-3, -7, and -9 wildtype, ER/PR positive, and p53 mutant [40]. The induction of apoptosis that occurs is possible via a p53-independent apoptotic mechanism, whereas MCF-7 cells are wildtype p53 and have a deletion in the *CASP-3* gene, so they do not express caspase-3 [41]. In cancer cells with p53 mutations, there will be a reduced response to agents that induce apoptosis, and thus the cancer may become resistant to antineoplastic drugs that target DNA damage. If p53 cannot bind to response elements in DNA, its ability to regulate the cell cycle can be reduced or lost. Conditions of cellular stress caused by ionizing chemotherapeutic agents or drugs that are genotoxic will result in damage to double-stranded or single-stranded DNA, which will then initiate the cell response to DNA damage [32]. DNA damage will initiate p53 to activate the cascade via the p21 pathway. If the DNA repair process cannot be carried out, p53 will activate other proapoptotic proteins such as Bax, Puma, Noxa, and Fs, which will initiate apoptosis [42]. If p53 activates the proapoptotic protein Bax and suppresses Bcl-2 expression, there will be an increase in mitochondrial permeability so that cytochrome c is released into the cytosol. Cytochrome c will bind to Apaf-1 to form a nucleosome complex, which will activate caspase-9 and initiate the caspase cascade, leading to cell death [43].

From the explanation above, compound (**1**) shows strong cytotoxic activity by increasing apoptosis and cell cycle modulation in the G1 phase of both MCF-7 and T47D cells. Further research is needed to identify the proteins involved in the molecular mechanisms underlying compound (**1**) through the expression of p53 protein, mutant p53, Bcl-2, and NF-Kß as well as through other signal transduction both in vitro and in vivo on cells breast cancer.

In this study, the cytotoxic activity of compound (**1**) against MCF-7 and T47D breast cancer cells was carried out in vitro and using a molecular docking approach. Using the docking approach, we tried to study molecularly the compound’s ability to inhibit ER and RP activity in breast cancer. Regarding anticancer activity, especially when using T47D cells, it is important to consider the crucial role of the estrogen receptor (ER) and the progesterone receptor (PR) because they regulate the growth and development of T47D cells [44]. In many cases, the ER and PR work synergistically in controlling T47D cell growth and differentiation. Estrogen often stimulates PR expression, thereby enhancing the effect of the hormone progesterone [45]. ERs can interact directly with PRs to generate protein complexes that co-regulate target gene expression [46].

The results showed that the compounds could inhibit the ER and PR with successive energies of −8.2 kcal/mol and −7.3 kcal/mol, respectively, which were better than doxorubicin. Interestingly, the hydroxyl group in compound (**1**) can form H-bonds with the Leu536 residue, which is considered crucial for the activity of ER [47]. Similarly, in the PR, the hydroxy group of this isolate contributes an H-bond that aids in binding to the crucial residue Arg766 in the PR’s ligand binding domain [48]. These simulation results align with our experiments, where, overall, compound (**1**) exhibited better activity than doxorubicin against T47D cells.

Caspase-9 is a key enzyme in the intrinsic apoptotic pathway and plays a role in initiating and regulating the process of apoptosis [49]. Caspase-9 activation is a critical step in activating the caspase pathway leading to the execution of apoptosis and programmed cell destruction [50]. Unlike the case with the ER and PR, which must be inhibited, for caspase-9, we tried to study the activation potential of caspase-9 from compound (**1**) to assess the ability to induce apoptosis of cancer cells. In this case, we mimicked the binding site of DTP (deoxyadenosine triphosphate), which is known to activate caspase-9 [51], so it is expected to provide an overview of caspase-9 activation from compound (**1**). Based on the simulation results, we identified that doxorubicin and compound (**1**) can activate caspase-9 with a strong affinity of −9.8 kcal/mol and −9.0 kcal/mol, respectively. The ability of compound (**1**) to interact with Mg^2+^ ions, which is a cofactor of caspase-9, plays a role in the affinity and activation of this target [50]. These results also correlate with our apoptosis experiments, where compound (**1**) and doxorubicin were found to trigger early apoptosis in cancer cells. In another study, doxorubicin was shown to promote mitochondrial-dependent apoptosis by inhibiting Bcl-xL regulation and the upregulation of Bax and caspase-9 expression [52]. In our analysis, the isolate’s hydroxyl groups were critical in interacting with all three targets (the ER and PR and caspase-9). These findings provide a fundamental contribution regarding the potential for the compounds to be developed as an anticancer drug.

## 4. Materials and Methods

### 4.1. General Procedure

Compound (**1**) was analyzed using a Fourier transform infrared spectrophotometer (FTIR 100 PERKIN ELMER) with KBr pellet, and the mass spectrum was recorded using LC-MS/MS waters Xevo G2-XS QTof with ionization type ESI. The ESI source was performed in positive ion mode. The ultraviolet (UV) spectrum was obtained in chloroform using a UV-Vis Hitachi UH5300 spectrophotometer. The ^1^H-(500 MHz), 13 C-(125 MHz), 1H-1H COSY, DEPT-135, HMQC, and HMBC nuclear magnetic resonance (NMR) spectra were identified using JEOL-ECZ500R with CDCl_3_. Silica gel 60 GF_254_ was used for preparative thin-layer chromatography (PLC), Silica gel 60 F_254_ for column chromatography, and TLC silica gel 60 F_254_ plates.

### 4.2. Plant Material

*Scoparia dulcis* Linn, native to tropical America, was naturalized in Java [53] (Backer & Bakhuizen van ddn Brink, 1965, Flora of Java, Vol. II, Wolters-Noordhoff N.V., Groningen, The Netherlands, p. 512) and grows wild in Indonesia. Sample plants were collected and harvested in the Kalasan area, Yogyakarta, Indonesia, in June 2020. Furthermore, plant determination was carried out based on the *Flora of Java* book No: BF/Ident/173/2017, where BF represents the Herbarium of the Department of Pharmaceutical Biology, Faculty of Pharmacy, Universitas Gadjah Mada, Yogyakarta, Indonesia, 173 indicates the collection number, and 2017 indicates samples collected in the year 2017. The determination was made using the reference book *Flora of Java*, Volumes I and II (Backer & van den Brink, 1962–1965). The book was used to determine plant species by comparing the characteristics inherent in a sample with the characteristics written in the book in a dichotomous manner.

### 4.3. Extraction and Separation

The powdered herb *Scoparia dulcis* Linn (6000 g) was macerated with 96% ethanol and then evaporated to obtain a thick extract. Then, the extract was triturated with n-hexane 3 L to produce a soluble fraction of n-hexane (FNH) (69.75 g) and insoluble n-hexane. The insoluble n-hexane fraction was then triturated again with ethyl acetate 3 L to produce a soluble ethyl acetate (FEA) fraction (23.52 g) and insoluble ethyl acetate. The insoluble fraction of n-hexane and ethyl acetate (precipitate) was 124.21 g. The FEA (20 g) was separated using vacuum liquid chromatography (VLC) with eluent gradient (n-hexane 100%; n-hexane-ethyl acetate 97:3; 90:10; 80:20; 70:30; to 100% ethyl acetate, and finally washed with MeOH). Subfractions were obtained by collecting subfractions every 100 mL. Furthermore, these subfractions were examined for their chromatogram profile using thin-layer chromatography (TLC) and tested for their cytotoxic activity against MCF-7 and T47D breast cancer cells. Then, they were selected for isolation based on the results of their cytotoxic activity against MCF-7 and T47D breast cancer cells. Then, subfraction 13 was selected to isolate the compound to obtain a pure compound. Isolation and purification of the compounds were carried out using thin-layer chromatography (TLC) and preparative thin-layer chromatography (PTLC). The pure compound (compound (**1**)) was tested for its cytotoxic activity against MCF-7 and T47D breast cancer cells and analyzed for structural elucidation using spectroscopy: UV-Vis, IR, LC-MS/MS, and NMR.

### 4.4. Thin-Layer Chromatography

The isolate was tested for spot chromatogram profiles with TLC silica gel 60 F_254_ plates using the appropriate mobile phases and for purity using three different mobile phases. The systems used for the isolate were n-hexane:ethyl acetate (2:3); chloroform:ethyl acetate (1:3); and n-hexane:ethanol (1:2). Spraying with vanillin sulfate was used to observe the spot chromatogram on the plates.

### 4.5. Cytotoxic Activity Assay

The cell lines were obtained from the Parasitology Laboratory, Faculty of Medicine, Universitas Gadjah Mada, Yogyakarta, Indonesia. Each MCF-7 and T47D cell was cultured with complete media, namely, DMEM media for MCF-7 and RPMI for T47D, which contained 10% FBS, 2% penicillin–streptomycin, and 0.5% fungzon. Cultures were stored in a 5% CO_2_ incubator and for harvesting cells using trypsin, as well as sample solvents using DMSO.

Compound (**1**) was tested for its cytotoxic activity using an MTT assay on MCF-7 and T47D breast cancer cells. Briefly, 1 × 10^4^ MCF-7 and T47D cells/wells were grown in 96 well plates and incubated for 24 h in a CO_2_ incubator. The cells were treated with sample concentrations of 200, 100, 50, 25, 12.5, and 6.25 μg/mL for MCF-7 and 50, 25, 12.5, 6.25, 3.125, and 1.5625 μg/mL for T47D and then incubated for 24 h in a CO_2_ incubator. After incubation, the condition of the cells was observed under a microscope. The cell media was discarded followed by washing with phosphate-buffered saline (PBS). Then 0.5 mg/mL MTT reagent, up to 100 µL, was added to each well followed by incubation again for 4 h in a CO_2_ incubator. The cells were observed under a microscope, and after the formazan was clear, 100 µL of SDS stopper reagent was added in 0.01 N HCl. The plate was incubated at room temperature in a dark place for 1 × 24 h, then the absorbance was read with a microplate reader at a wavelength of 595 nm [54]. Analysis of the absorbance data for the cytotoxic test was carried out by calculating the percentage of viable cells using the following formula. Doxorubicin was used as a positive control.

$$\%\;\mathrm{Cell}\;\mathrm{Viability}=\frac{Absorbance\;(treatment-media\;control)}{Absorbance\;(Cell\;control-media\;control)}\times 100$$

IC_50_ values were calculated using a linear regression between sample concentrations and the percent (%)cell viability.

### 4.6. Cell Cycle and Apoptosis Assay

This test was carried out on isolated samples (compound (**1**)). Culture confluent cells were distributed to 6-well plates, each 2000 µL (for treatment and for cell control). After 24 h of incubation, concentration series were made. The cells were washed with PBS, and 1000 µL of the sample solution with a certain concentration series was put in the well. For the control cells, 1000 µL of culture medium was added well. The cells were again incubated for 24 h in the CO incubator. After incubation, media from the 6-well plates were inserted into a conical tube, then washed with 1000 µL PBS and put into the same conical tube. Then, 200 µL trypsin-EDTA 0.25% was added and incubated for 3 min. Then, 1000 µL of culture medium was also added and resuspended until the cells were released one by one. The cells are then transferred to a conical and centrifuged at 2000 rpm for 3 min. For cell cycle observations, a deep cell precipitates conical tube covered with aluminum foil was dissolved with 400 µL of the PI reagent containing 1 mg/mL PI, 10 mg/mL RNAse, and 0.1% (*v*/*v*) Triton-X 100. The cells were resuspended and incubated for 5 min, and then the cell suspension was transferred into a flow cytometry tube for analysis (Ayers et al., 2011). For observations of apoptosis, the supernatant was discarded, then 100 µL of Annexin-V-FLUOS staining was added consisting of 100 µL binding buffer, 2 µL Annexin V, and 2 µL PI. This was followed by incubation in a dark space for 10 min and then analysis using a flow cytometer [55].

### 4.7. Molecular Docking Study

To evaluate the anticancer and apoptotic activities of isolated compounds molecularly, we used crystal structures of estrogen receptor (ER) (PDB ID 3ERT) [47], progesterone receptor (PR) (PDB ID 1E3K) [48], and caspase-9 (PDB ID 5WVE) [51].

The receptors were prepared by eliminating water molecules and bound ligands from their crystal structures. In AutoDock Tools v1.5.6, the target protein was protonated by adding polar hydrogen atoms and Kollman charges [56]. Meanwhile, the structures of the isolated compound and doxorubicin were drawn using ChemDraw [57] and optimized in HyperChem with semi-empirical AM1 methods [58]. Lastly, hydrogen atoms were added to the compound structures, and the Gasteiger charges were adjusted using AutoDock Tools v1.5.6 [59].

The binding affinity and interactions of the isolated compound were determined using AutoDock Vina [60]. The co-crystal ligands (TMX, R18, and DTP) were redocked to the ER and PR and caspase-9, respectively, to validate the docking parameters. The validated procedures were identified with a root mean square deviation (RMSD) below 2 Å [61]. The binding sites on the ER and PR and caspase-9 were set according to their native ligand positions and were calculated using the cubic shape with a grid area of 30 × 30 × 30 Å. The other docking procedures were set as the default. Lastly, the protein and compound interactions were analyzed and visualized with help of Discovery Studio Visualizer version v.17.2.0.16349 software.

## 5. Conclusions

A new compound isolated from the ethyl acetate fraction of *S. dulcis* Linn was identified as 2α-hydroxyscopadiol. This compound has never been reported in *S. dulcis* Linn, and it shows strong and very strong cytotoxic activities in MCF-7 and T47D breast cancer cells, respectively, and has the ability to inhibit the cell cycle and induce apoptosis of both cells. Molecularly, this compound can inhibit the activity of the ER and PR and induce apoptosis through the activation of caspase-9. These findings provide a fundamental contribution regarding the potential of the isolate from *S. dulcis* to be developed as an anticancer agent.

## Figures and Tables

**Table 1:** The ^1^H NMR spectral data for compounds (**1**) ^a^, (**2**), (**3**) [22], (**4**) [23], and (**5**) [21].

^1^H (δppm)	1	2	3	4	5
H-1	1.13 m; 1.58 m	1.76 m; 1.19 br d (13.9)	1.82 m; 1.29 br d (13.3)	-	-
H-2	4.20 m	1.75 m; 1.58 m	1.77m; 1.67 m	-	-
H-3	1.63 m	1.73 m; 1.21 br d (13.6)	1.86 m; 1.77 m	-	-
H-5	2.06 d (2.5)	2.47 br d (2.2)	2.88 d (2.0)	2.06 br d (2.4)	2.06 br d (2.3)
H-6	5.60 s	5.51 t (2.4)	5.30 t (2.4)	5.63 br d (2.4)	5.63 br d (2.0)
H-7	2.33 m	4.16 d (2.4)	5.29 d (2.5)	2.40 m	2.36 m
H-9	1.80 m	1.88 br s	1.97 m	-	-
H-11	1.74 m	1.91 dd (10.6; 4.1); 1.77 m	1.81 m	-	-
H-12	1.83 m	2.11 br t (11.1); 1.85 m	1.96 m; 1.83 m	-	-
H-14	5.12 d (10)	5.43 t (6.9)	5.43 t (6.9)	5.42 br t (6.8)	5.35 br t (7.0)
H-15	3.66 m; 3.50 dt (5;10)	4.13 d (6.9)	4.19 d (6.9)	4.16 d (6.8)	4.60 d (7.0)
H-16	1.24 s	1.68 s	1.71 s	1.68 s	1.71 s
H-17	4.71 s	5.03 d (2.1); 4.90 d (2.1)	5.26 d (1.8); 5.07 d (1.8)	4.73 s	4.73 s
H-18	3.59 d (11); 3.10 d (11)	3.66 d (11.4); 3.10 d (11.4)	1.35 s	3.60 d (11.0); 3.15 d (11.0)	3.61 d (10.9); 3.16 d (10.6)
H-19	0.88 s	0.89 s		0.91 s	0.92 s
H-20	1.44 s	1.43 s	1.49 s	1.46 s	1.47 s
H-2′6′	8.01 d (7.5)	8.01 d (7.4)	8.03 d (7.4)	8.04 d (7.3)	8.04 d (7.6)
H-3′5′	7.43 t (7.5)	7.45 t (7.4)	7.46 t (7.4)	7.44 t (7.3)	7.45 t (7.6)
H-4′	7.54 t (7.5)	7.57 t (7.4)	7.58 t (7.4)	7.56 t (7.4)	7.56 t (7.6)
H-2″	-	-	2.09 s	-	2.31 t (7.2)
H-3″-9″	-	-	-	-	1.2 –1.70 18 H
H-10″	-	-	-	-	0.90 t (7.2)

^a^ Spectra recorded at 500 MHz (^1^H NMR (ppm)) in CDCl_3_.

**Table 2:** The ^13^C NMR spectral data for compounds (**1**) ^a^, (**2**), (**3**) [22], (**4**) [23], and (**5**) [21].

^13^C	1	2	3	4	5
C-1	38.1	38.7	38.3	38.7	38.7
C-2	75.2	18.5	18.2	18.7	18.8
C-3	38.1	38.0	40.2	38.6	38.6
C-4	38.8	38.6	46.8	38.3	38.3
C-5	41.1	35.9	38.9	41.5	41.6
C-6	71.7	74.7	73.9	71.8	71.8
C-7	36.9	75.7	75.2	37.1	37.1
C-8	144.4	145.2	140.4	144.5	144.5
C-9	57.5	56.6	56.7	57.6	57.6
C-10	38.5	38.4	38.2	38.9	38.9
C-11	24.7	26.6	26.0	24.4	24.4
C-12	31.5	38.7	38.2	38.3	38.3
C-13	148.9	140.6	140.0	140.3	142.6
C-14	111.0	123.1	123.1	123.4	118.6
C-15	65.8	59.4	59.4	59.6	61.4
C-16	29.8	16.6	16.7	16.8	16.9
C-17	113.2	118.3	121.9	113.2	113.2
C-18	71.2	70.8	182.1	71.5	71.5
C-19	20.5	20.9	19.7	20.5	20.5
C-20	26.0	26.0	25.7	26.1	26.1
C=O	166.4	166.4	165.6	166.5	166.5
C-1′	130.7	130.3	130.0	131.0	131.0
C-2′6′	129.7	129.7	129.8	129.9	129.9
C-3′5′	128.5	128.5	128.5	128.7	128.7
C-4′	132.9	133.1	133.2	133.1	133.1
C-1″	-	-	21.3	-	174.1
C-2″	-	-	-	-	38.4
C-3″-9″	-	-	-	-	25.1–38.9
C-10″	-	-	-	-	15.0

^a^ Spectra recorded at 125 MHz (^13^C NMR (ppm)) in CDCl_3_.

**Table 3:** Cytotoxic activity of compound (**1**) and doxorubicin against MCF-7 and T47D breast cancer cells and Vero cells.

NO	Sample	IC_50_ (μg/mL)			Selectivity Index (SI)	
		MCF-7	T47D	Vero	MCF-7	T47D
1	Compound (1)	70.56 ± 1.54 ^a^	<3.125 ± 0.43 ^a^	36.50 ± 1.41 ^a^	0.51	12.67
2	Doxorubicin	16.62 ± 5.85 ^b^	35.08 ± 3.14 ^b^	69.52 ± 6.80 ^b^	4.2	1.45

The IC_50_ values are represented as mean ± standard deviation (SD). Different letters (^a^,^b^) indicate significant differences based on an ANOVA followed by a *t*-test (*p* < 0.05).

**Table 4:** Summary of docking results for the isolate to the ER and PR and caspase-9.

Compound	Binding Energy (kcal/mol)		
	Estrogen Receptor (PDB ID 3ERT)	Progesterone Receptor (PDB ID 1E3K)	Caspase-9 (PDB ID 5WVE)
TMX (Tamoxifen)	−9.8	-	-
R18	-	−11.5	-
DTP	-	-	−9.9
2α-hydroxyscopadiol	−8.2	−7.3	−9.0
Doxorubicin	−7.7	−5.6	−9.8

## Data Availability

The data are contained within this article or the Supplementary Materials.

## Supplementary Materials

The following supporting information can be downloaded at: https://www.mdpi.com/article/10.3390/molecules28165960/s1, NMR and LC-MS/MS spectra of the new compound, ^1^H and ^13^C NMR data on 2α-hydroxyscopadiol, key acquisition parameters of COSY, HMBC, and NOESY spectra, and molecular docking for compound **1**.
