# Supplementary material for: A New Diterpenoid of Indonesian Scoparia dulcis Linn: Isolation and Cytotoxic Activity against MCF-7 and T47D Cell Lines
Source: Molecules. 2023 Aug 9;28(16):5960. doi: 10.3390/molecules28165960 (PMC10459870; doi:10.3390/molecules28165960)
Supplement: Supplementary file 1 [file molecules-28-05960-s001.zip › molecules-2506131-supplementary.pdf]

Supplementary Materials

A New Diterpenoid of Indonesian *Scoparia dulcis* Linn: Isolation and Cytotoxic Activity against MCF-7 and T47D Cell Lines

Hasnawati Hasnawati [1,2,*], Subagus Wahyuono [3,*], Ratna Asmah Susidarti [4], Djoko Santosa [3] and Arfan Arfan [1]

[1] Faculty of Pharmacy, Universitas Halu Oleo, Kendari 93232, Indonesia; arfan09@uho.ac.id
[2] Doctoral Program in Pharmaceutical Science, Faculty of Pharmacy, Universitas Gadjah Mada, Yogyakarta 55281, Indonesia
[3] Department of Pharmaceutical Biology, Faculty of Pharmacy, Universitas Gadjah Mada, Yogyakarta 55281, Indonesia; djoko5346@ugm.ac.id
[4] Department of Chemistry Pharmaceutical, Faculty of Pharmacy, Universitas Gadjah Mada, Yogyakarta, 55281, Indonesia; ratna_asmah@ugm.ac.id
* Correspondence: hasna@uho.ac.id (H.H.); subagusw_fa@ugm.ac.id (S.W.)

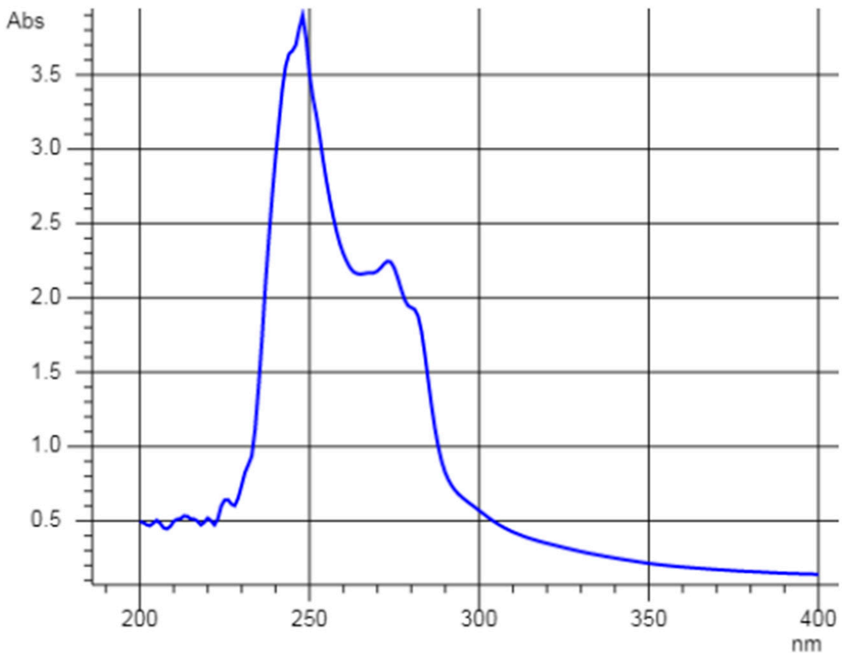

Figure S1. UV-Vis spectra of Compound (**1**) (λ_{max}).

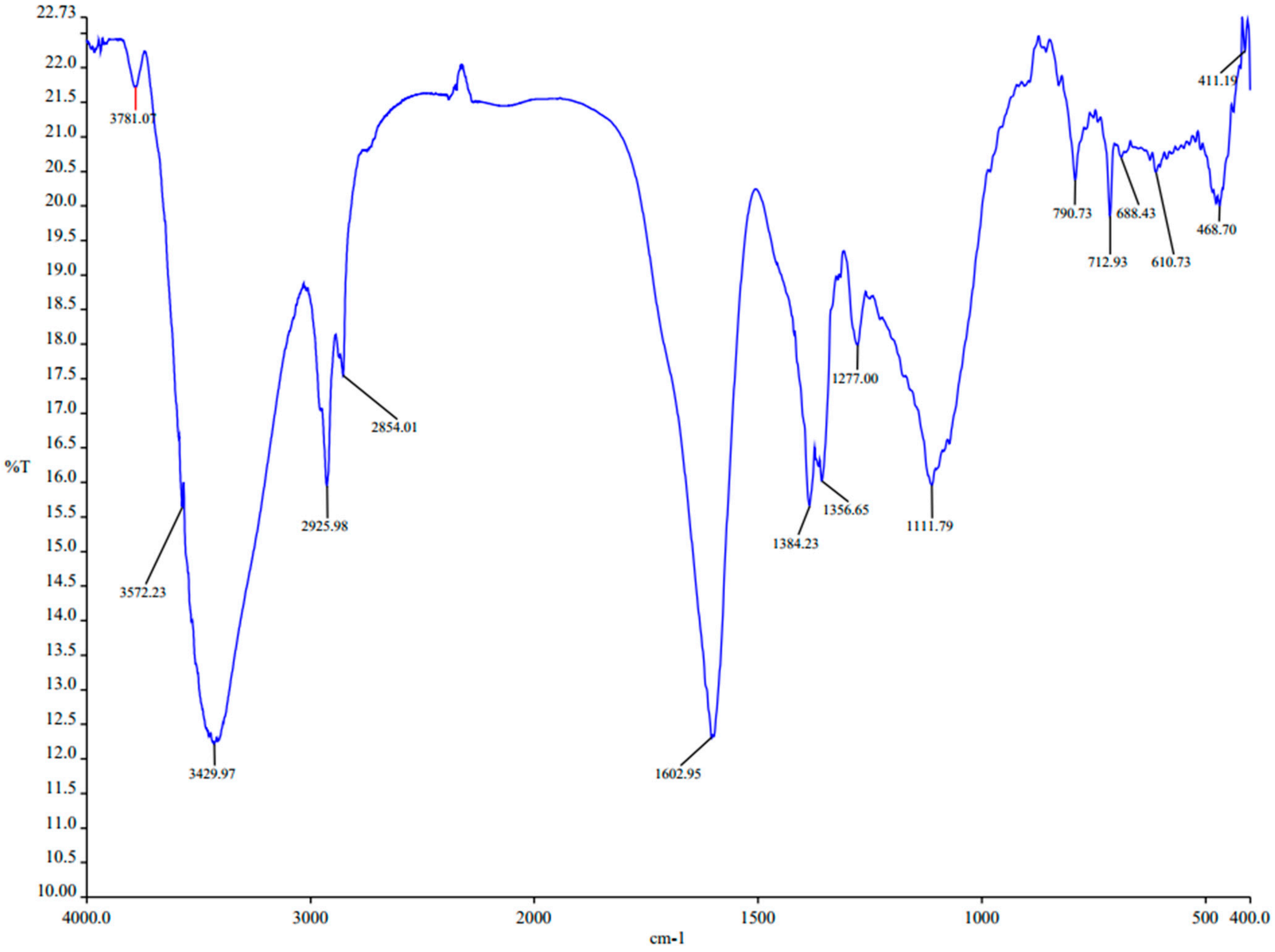

Figure S2. IR spectra of Compound (**1**).

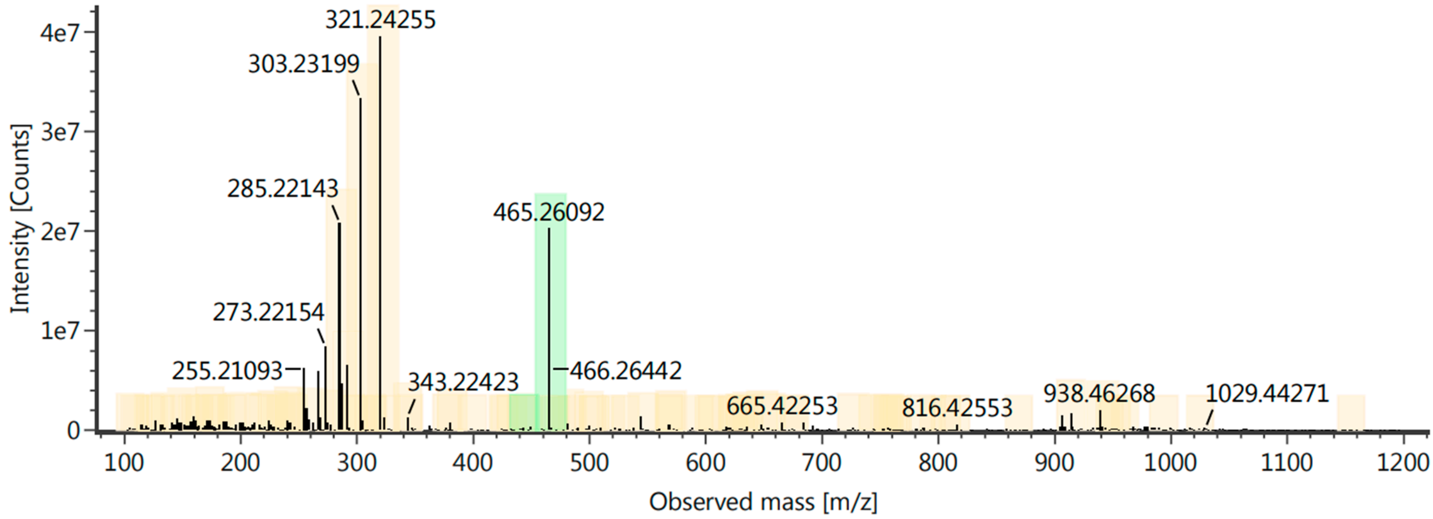

Figure S3. LC-MS/MS spectra of compound (**1**).

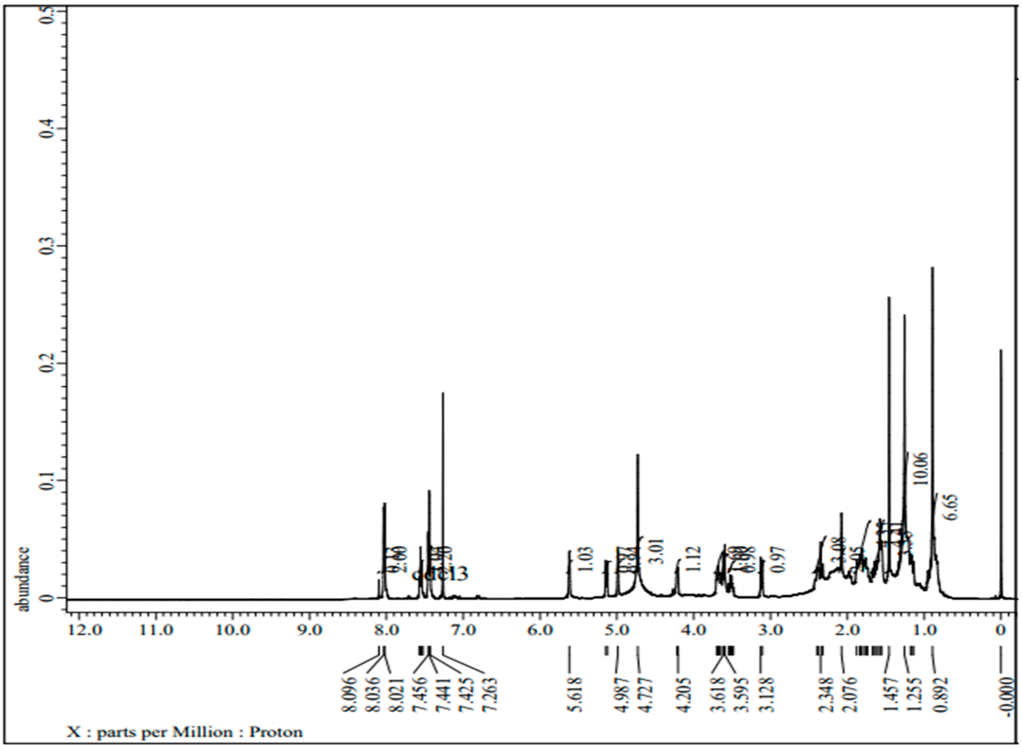

Figure S4. ^{1}H - NMR spectra of Compound (**1**).

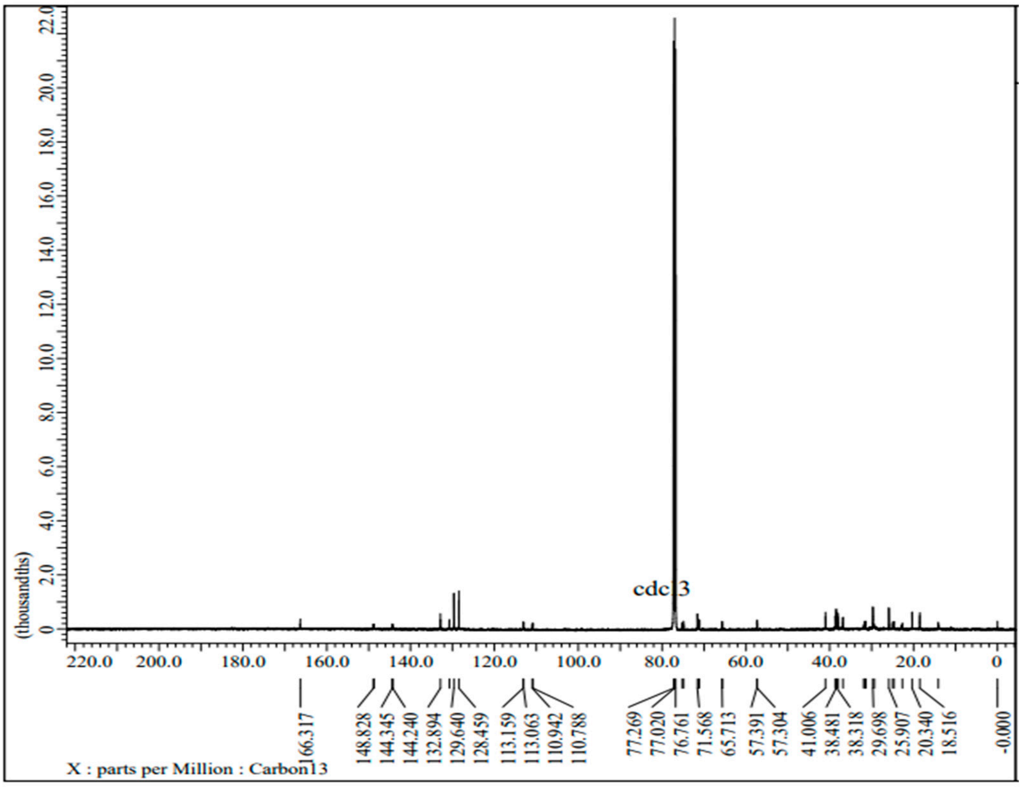

Figure S5. ^{13}C - NMR spectra of Compound (**1**).

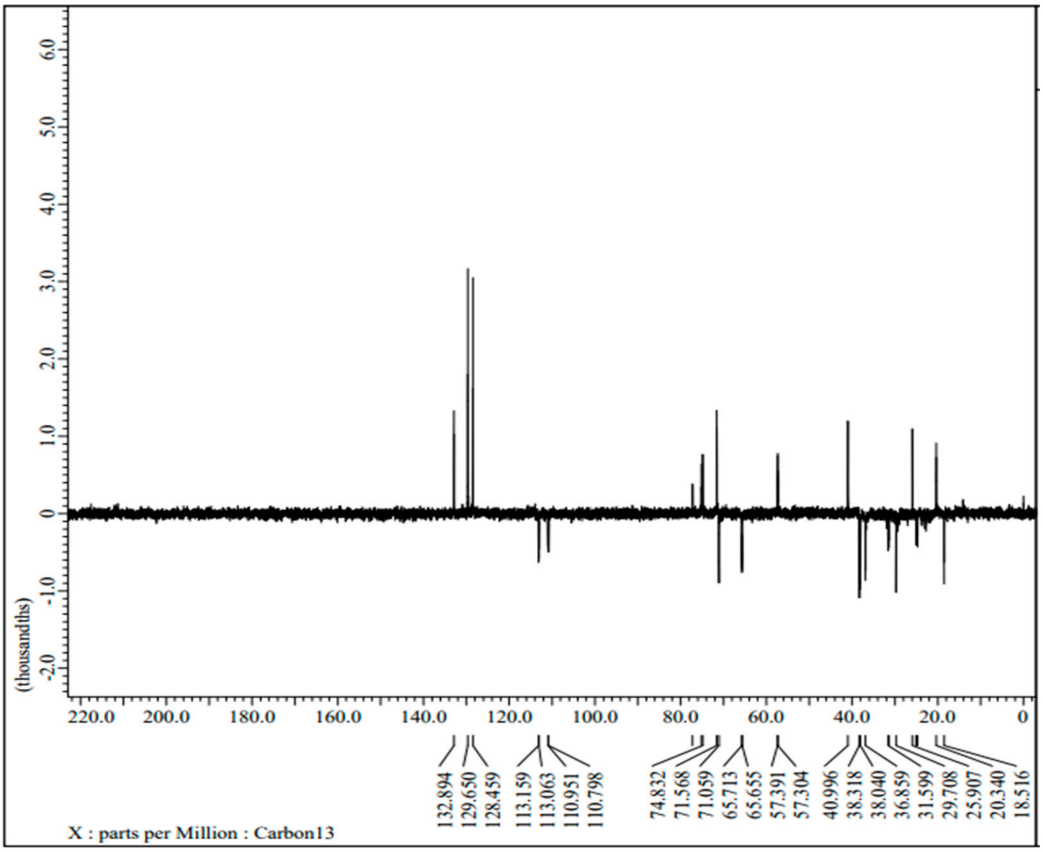

Figure S6. DEPT-135 spectra of Compound (**1**).

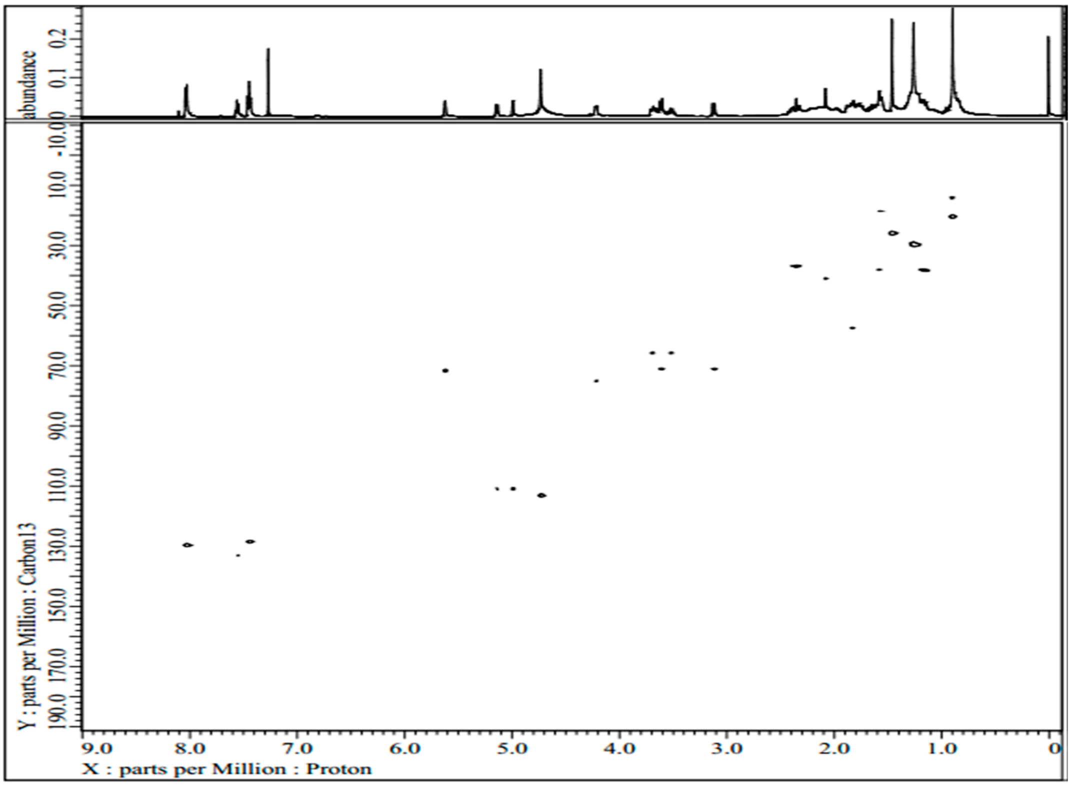

Figure S7. HSQC spectra of Compound (**1**).

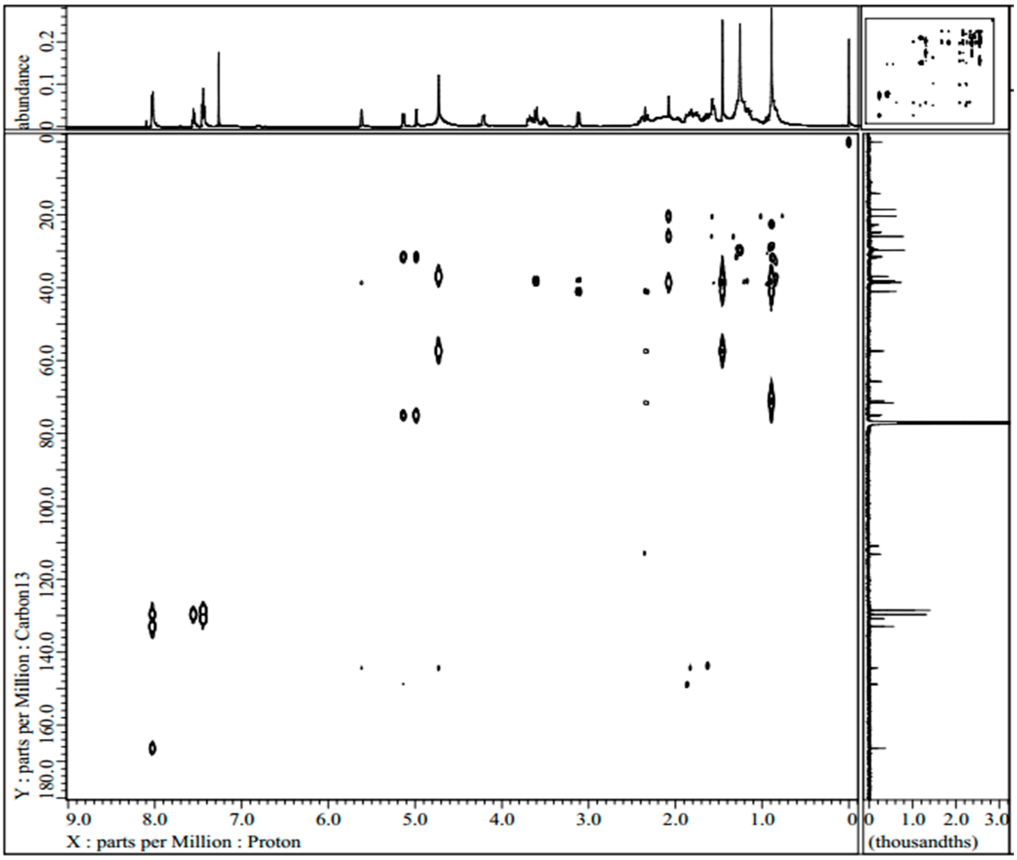

Figure S8. HMBC spectra of Compound (**1**).

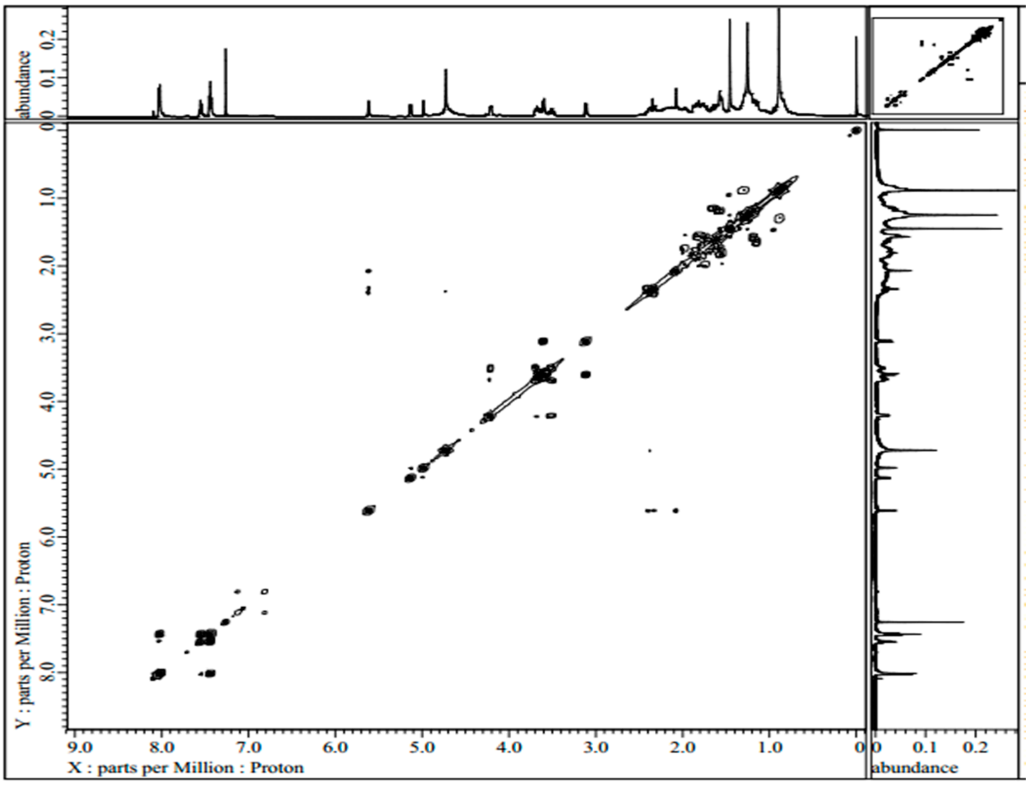

Figure S9. COSY spectra of Compound (**1**).

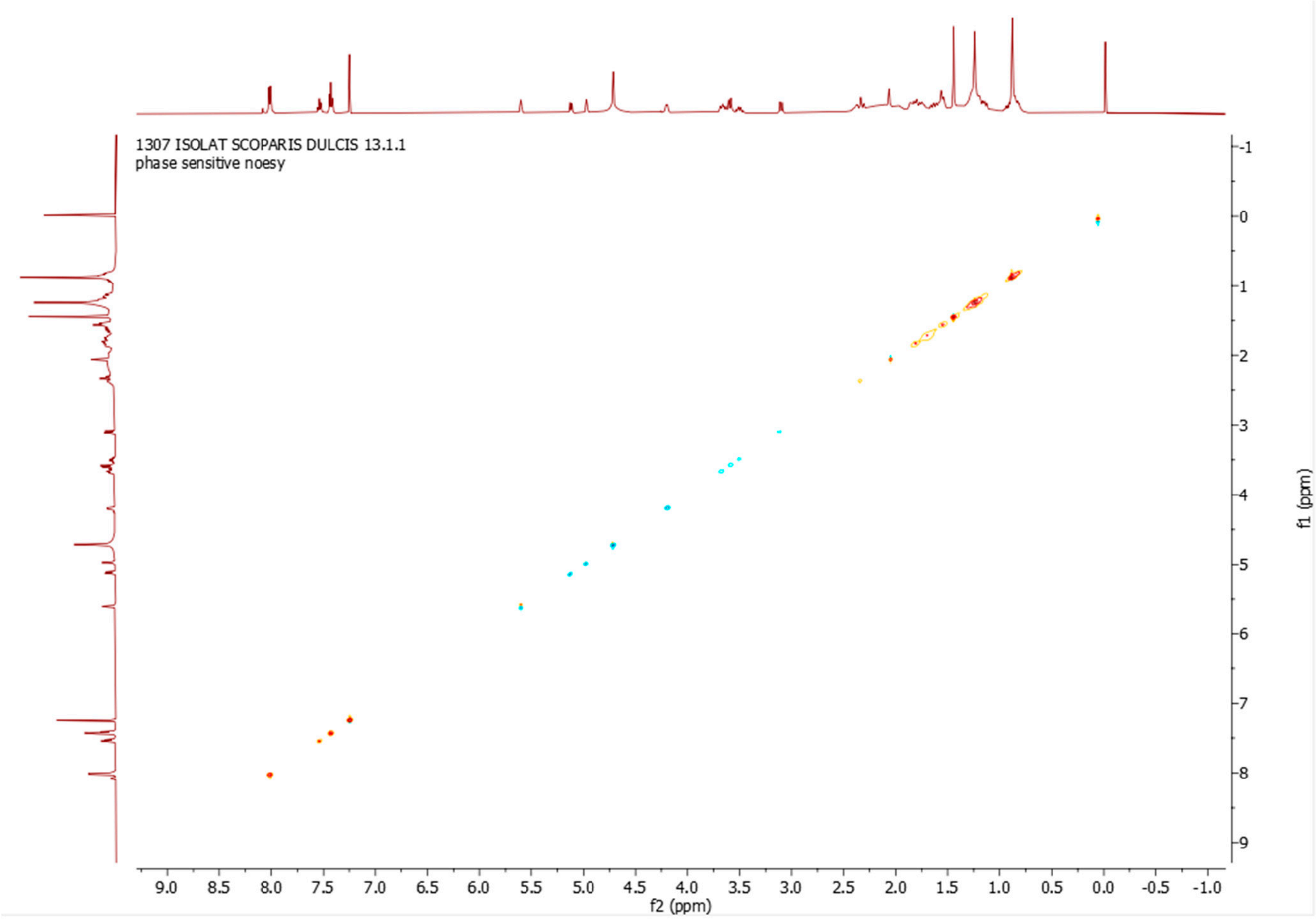

Figure S10. NOESY spectra of Compound (**1**).

(**a**) (**b**) (**c**)

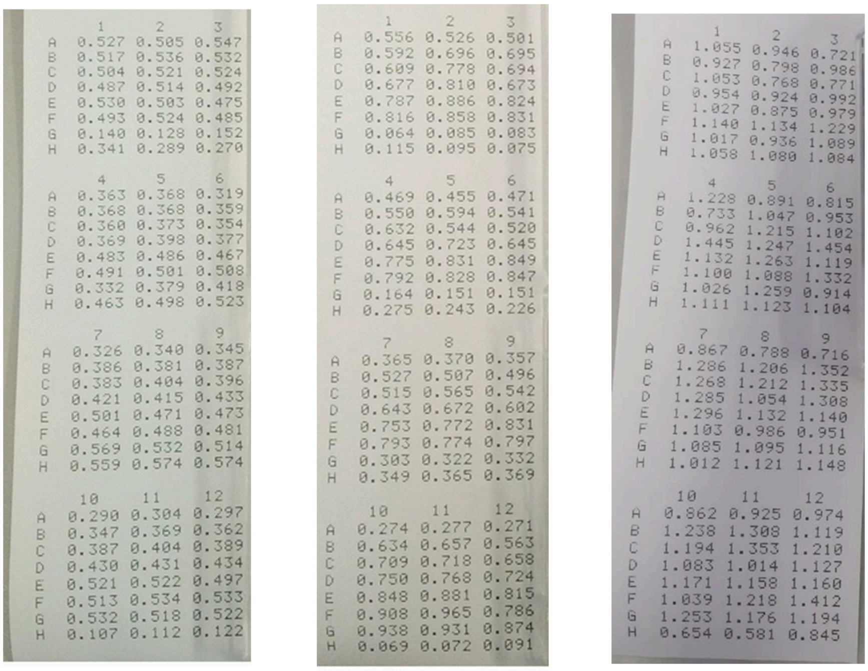

(a)

	1	2	3	4	5	6	7	8	9	10	11	12
A	0.527	0.505	0.547	0.363	0.368	0.319	0.326	0.340	0.345	0.290	0.304	0.297
B	0.517	0.536	0.532	0.368	0.368	0.359	0.386	0.381	0.387	0.347	0.369	0.362
C	0.504	0.521	0.524	0.360	0.373	0.354	0.383	0.404	0.396	0.387	0.404	0.389
D	0.487	0.514	0.492	0.369	0.398	0.377	0.421	0.415	0.433	0.430	0.431	0.434
E	0.530	0.503	0.475	0.483	0.486	0.467	0.501	0.471	0.473	0.521	0.522	0.497
F	0.493	0.524	0.485	0.491	0.501	0.508	0.464	0.488	0.481	0.513	0.534	0.533
G	0.140	0.128	0.152	0.332	0.379	0.418	0.569	0.532	0.514	0.532	0.518	0.522
H	0.341	0.289	0.270	0.463	0.498	0.523	0.559	0.574	0.574	0.107	0.112	0.122

(b)

	1	2	3	4	5	6	7	8	9	10	11	12
A	0.556	0.526	0.501	0.469	0.455	0.471	0.365	0.370	0.357	0.274	0.277	0.271
B	0.592	0.696	0.695	0.550	0.594	0.541	0.527	0.507	0.496	0.634	0.657	0.563
C	0.609	0.778	0.694	0.632	0.544	0.520	0.515	0.565	0.542	0.709	0.718	0.658
D	0.677	0.810	0.673	0.645	0.723	0.645	0.643	0.672	0.602	0.750	0.768	0.724
E	0.787	0.886	0.824	0.775	0.831	0.849	0.753	0.772	0.831	0.848	0.881	0.815
F	0.816	0.858	0.831	0.792	0.828	0.847	0.793	0.774	0.797	0.908	0.965	0.786
G	0.064	0.085	0.083	0.164	0.151	0.151	0.303	0.322	0.332	0.938	0.931	0.874
H	0.115	0.095	0.075	0.275	0.243	0.226	0.349	0.365	0.369	0.069	0.072	0.091

(c)

	1	2	3	4	5	6	7	8	9	10	11	12
A	1.055	0.946	0.721	1.228	0.891	0.815	0.867	0.788	0.716	0.862	0.925	0.974
B	0.927	0.798	0.986	0.733	1.047	0.953	1.286	1.206	1.352	1.238	1.308	1.119
C	1.053	0.768	0.771	0.962	1.215	1.102	1.268	1.212	1.335	1.194	1.353	1.210
D	0.954	0.924	0.992	1.445	1.247	1.454	1.285	1.054	1.308	1.083	1.014	1.127
E	1.027	0.875	0.979	1.132	1.263	1.119	1.296	1.132	1.140	1.171	1.158	1.160
F	1.140	1.134	1.229	1.100	1.088	1.332	1.103	0.986	0.951	1.039	1.218	1.412
G	1.017	0.936	1.089	1.026	1.259	0.914	1.085	1.095	1.116	1.253	1.176	1.194
H	1.058	1.080	1.084	1.111	1.123	1.104	1.012	1.121	1.148	0.654	0.581	0.845

Figure S11. MTT assay (Absorbance cell control and absorbance of Compound (**1**)) against MCF-7 (a) and T47D (b) and (Absorbance doxorubicine against MCF-7, T47D, vero and compound (1) against vero cell) (c).

Cell cycle inhibition and apoptosis induction tests were carried out on compound (**1**). The analysis was performed using the flow cytometry method to determine the percentage of living cells in each phase of the cell cycle.

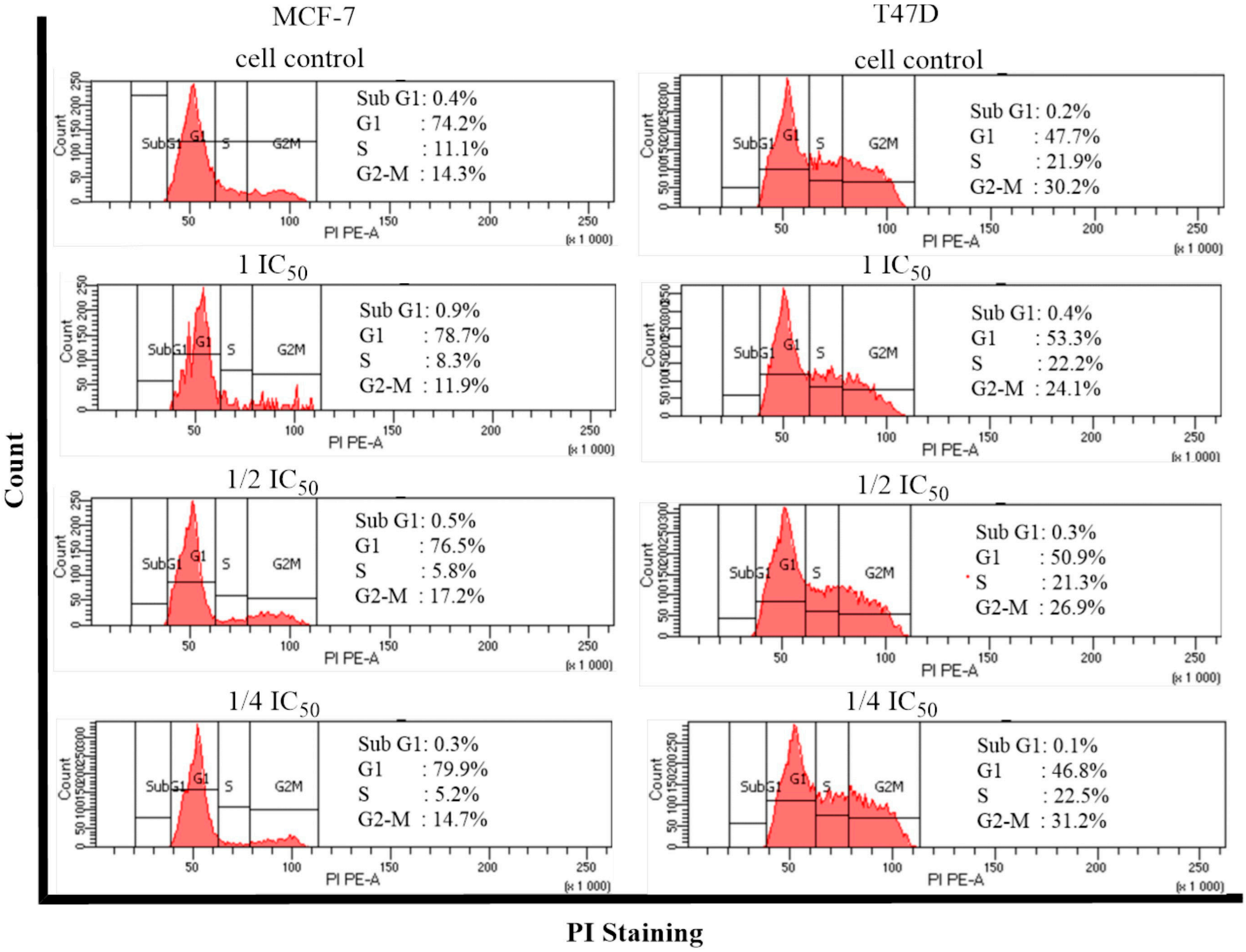

Figure S12. Histogram profile of the cell cycle for compound (**1**) against MCF-7 and T47D breast cancer cells and the control.

Induction of apoptosis was observed to detrmine the cell mechanism underlying the treatment of MCF-7 and T47D breast cancer cells with compound (**1**), which were incubated for 24 h. The method used in this study was the detected Annexin V method using flow cytometry, which allowed for observing the induction of apoptosis that occurs in cells following treatment. Annexin V is a member of a family of phospholipid-binding proteins that are strongly negatively charged on the cell membrane. Cell death resulting from apoptosis or necrosis can be differentiated using propidium iodide (PI) staining intercalation with DNA.

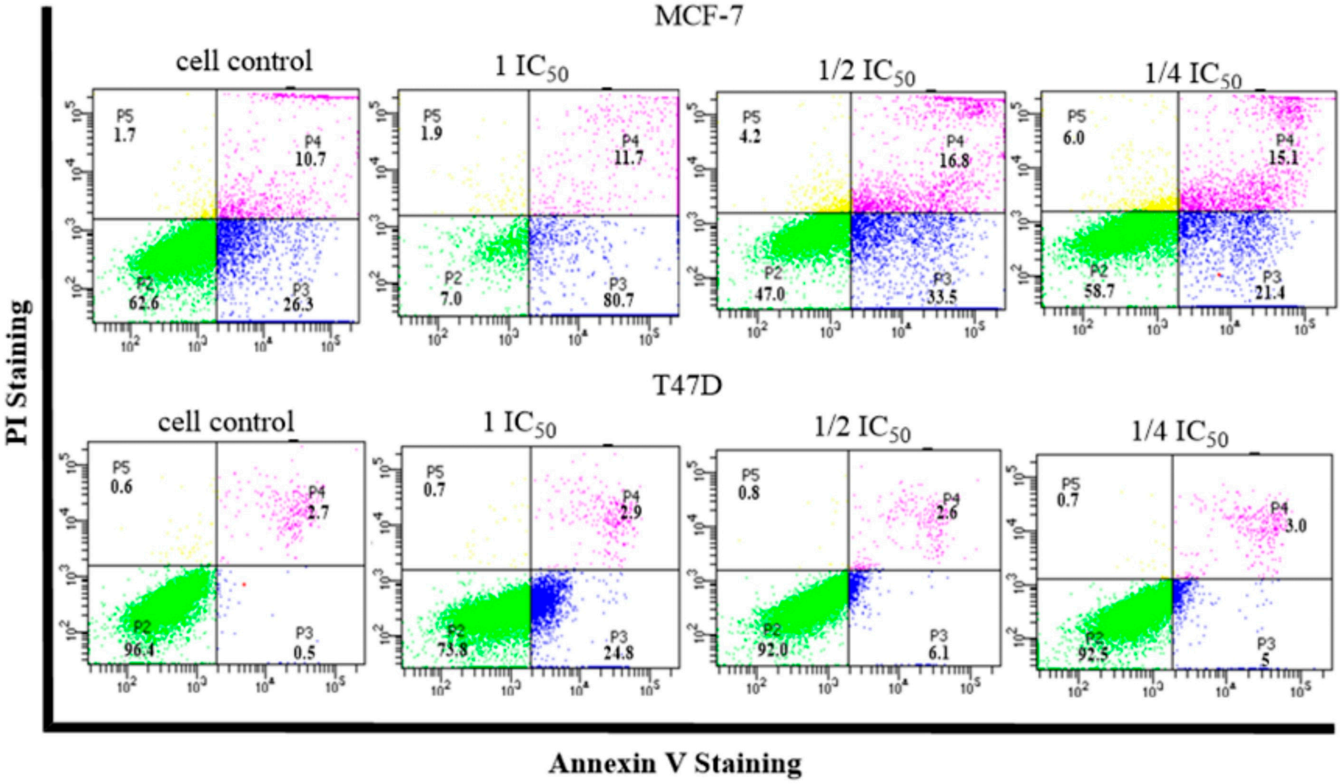

Figure S13. Histograme profile of apoptosis compound (1) to MCF-7, T47D breast cancer cell and cell control.
